# Lectin-Fc(IgG2a) fusion proteins binding to the cell wall of *C. albicans* cause structural, metabolic, oxidative, and other pleiotropic effects

**DOI:** 10.1128/spectrum.03645-25

**Published:** 2026-05-14

**Authors:** Susana Ruiz Mendoza, Claudia Rodriguez de La Noval, Marina da Silva Ferreira, Leandro Honorato, Jhon Jhamilton Artunduaga Bonilla, Luis Felipe Costa Ramos, Gilberto Domont, José Mauro Peralta, Leonardo Nimrichter, Allan Jefferson Guimarães

**Affiliations:** 1 Laboratório de Bioquímica e Imunologia das Micoses, Instituto Biomédico, Fluminense Federal University 28110, Niterói, Brazil; 2 Pós-Graduação em Imunologia e Inflamação, Instituto de Microbiologia Paulo de Góes, Federal University of Rio de Janeiro 28125, Rio de Janeiro, Brazil; 3 Laboratório de Glicobiologia de Eucariotos, Instituto de Microbiologia Paulo de Góes, Federal University of Rio de Janeiro 28125, Rio de Janeiro, Brazil; 4 Laboratório de Química de Proteínas, Departamento de Bioquímica, Instituto de Química, Universidade Federal do Rio de Janeiro 28125, Rio de Janeiro, Brazil; 5 Rede Micologia RJ – Fundação de Amparo à Pesquisa do Estado do Rio de Janeiro (FAPERJ), Rio de Janeiro, Brazil; 6 Pós-Graduação em Microbiologia e Parasitologia Aplicadas, Instituto Biomédico, Fluminense Federal University 28110, Niterói, Brazil; 7 National Institute of Science and Technology (INCT) in Human Pathogenic Fungi, São Paulo, Brazil; Institut Pasteur, Paris, France

**Keywords:** lectin-fusion proteins, chimeric proteins, antifungals, biopharmaceuticals, passive immunization, chitin, β-glucan, lectin-Fc(IgG2a) proteins

## Abstract

*Candida albicans,* an opportunistic fungal pathogen and the leading cause of hospital-acquired infections, exhibits diverse virulence factors that promote its survival within the human body. The rising incidence of multidrug resistance (MDR) in *C. albicans* requires urgent development of novel antifungal strategies. Through genetic engineering, our group developed Lectin-Fc(IgG2a) proteins [WGA-Fc(IgG2a) and Dectin-1-Fc(IgG2a)], which exhibit potent antifungal activity against multiple fungal pathogens and hold promise as broad-spectrum therapeutic agents. In this study, we investigated the direct effects of Lectin-Fc(IgG2a) fusion proteins on *C. albicans*, uncovering multiple mechanisms that compromise fungal viability. Notably, these antibody-like structures were only partially degraded by secreted aspartyl proteases (Saps) *in vitro*, as *sap* mutants exhibited increased susceptibility to growth inhibition. These proteins decreased key fungal cell wall polysaccharides, induced oxidative stress, and disrupted yeast intracellular micronutrient and lipid homeostasis. Furthermore, Lectin-Fc(IgG2a) treatment impaired secretory pathways and significantly reduced the release of extracellular vesicles. Proteomic analysis revealed marked changes in the protein expression profiles of *C. albicans*, including the enhanced expression of proteins related to stress response and metabolic shift. Moreover, Lectin-Fc(IgG2a) opsonization enhanced dendritic cell activation, contributing to fungal killing. Collectively, these findings support the antifungal direct effects and therapeutic potential of Lectin-Fc(IgG2a) proteins. In addition to their antibody-like functions, they alter fungal morphological features and induce oxidative and metabolic stress, increasing the susceptibility of their cell wall to host immune recognition and clearance.

**IMPORTANCE:**

*Candida albicans* is a major cause of life-threatening infections, particularly in hospitalized and immunocompromised individuals, and is increasingly resistant to available antifungal drugs. This study described the effects of a novel immunotherapeutic strategy using genetically engineered antibody-like fusion proteins—Lectin-Fc(IgG2a)—that directly impact fungal growth, disrupt cell wall integrity, induce oxidative stress, and suppress extracellular vesicle production, while also boosting immune cell activation. By targeting multiple fungal vulnerabilities simultaneously, these fusion proteins offer a promising alternative to traditional antifungals as demonstrated *in vivo* in previous reports by our group, with potential to reshape antifungal therapy and address the global threat of drug-resistant fungal infections.

## INTRODUCTION

Human candidiasis is caused by the opportunistic fungi *Candida* spp., with at least 15 recognized species identified as potential human pathogens. The majority of invasive infections are attributed to five main species: *C. albicans*, *C. glabrata* (recently renamed as *Nakaseomyces glabratus* [1], *C. tropicalis*, *C. parapsilosis*, and *C. krusei* (recently named as *Pichia kudriavzevii* [2, 3]). More recently, *C. auris* (recently renamed as *Candidozyma auris* [4]), a previously uncommon organism, has emerged as a major global health concern due to its rapid spread and resistance profile (5). *C. albicans* is a common commensal organism that colonizes the skin, gastrointestinal tract, and mucosal surfaces of approximately 30%–50% of healthy individuals (6). However, in immunocompromised individuals, clinical presentations may range from superficial and mucosal to systemic candidiasis (3, 7), with the latter associated with mortality rates as high as 60% (3, 6). Among cases of invasive candidiasis, *C. albicans* remains the prominent causative species, accounting for 46.3% of infections, followed by *C. glabrata* (24.4%) and *C. parapsilosis* (8.1%) (8). *Candida* species are particularly problematic in intensive care unit (ICU) and pediatric hospital settings, where they contribute to chronic infections and substantial healthcare expenses (9, 10).

*C. albicans* exhibits remarkable morphological plasticity, transitioning between unicellular yeast, pseudo-hyphal structures, and tissue-invading filamentous forms. These distinct morphotypes are accompanied by a repertoire of virulence traits that facilitate nutrient acquisition and enable adaptation within the host (6, 11). In fact, *C. albicans* yeast-to-hyphae morphological transition, along with the secretion of hydrolytic enzymes, is crucial for tissue invasion and pathogenesis (12). Filamentation, in addition to the expression of adhesins, contributes to biofilm formation, a key virulence attribute that protects the fungus from host immune responses and antifungal agents (13, 14). More recently, the release of extracellular vesicles (EVs) has emerged as a mechanism of fungal unconventional secretion and pathogenesis (15, 16). These EVs function as specialized compartments, carrying lipids, proteins, polysaccharides, pigments, RNA, and nucleic acids, and are implicated in growth regulation, morphogenesis, and host-pathogen interactions. Remarkably, these EVs can traverse the cell wall, contributing to biofilm formation and modulating host cell function and immune responses (17–19).

The treatment of fungal infections remains challenging due to several factors: (i) difficulties in accurately identifying the causative agent, (ii) the prolonged antifungal regimens, (iii) the emergence of resistance, and (iv) significant toxicity and side effects, including an often-associated enhanced inflammatory response (20, 21). The global rise of antifungal resistance has further narrowed the portfolio of effective treatment options. Certain *Candida* strains exhibit resistance to first-line antifungals such as echinocandins and fluconazole (22), while multidrug-resistant (MDR) and even extensively drug-resistant (XDR) strains are increasingly reported (23–25). Consequently, the development of novel antifungal strategies is urgently needed. Among emerging approaches, monoclonal antibody (mAb)-based therapeutics have shown promise, particularly against MDR fungi (26, 27).

Our group recently developed Lectin-Fc(IgG2a) fusion proteins with antibody-like properties, combining the fungal cell wall polysaccharide-binding domains of lectins with the effector region (Fc) of murine immunoglobulin G (IgG). Specifically, wheat germ agglutinin (WGA)-Fc(IgG2a) selectively binds chitoligomers, while Dectin-1-Fc(IgG2a) displays high affinity for β-1,3-glucan. Since both polysaccharides are universally expressed in the cell walls of various pathogenic fungal species, these offer the advantage of broad-spectrum application, potentially allowing earlier intervention, prior to a species-specific diagnosis (28). Previous studies have shown that Lectin-Fc(IgG2a) proteins exerted direct antifungal activity against *Histoplasma capsulatum*, *Cryptococcus neoformans*, *Aspergillus fumigatus, C. albicans,* and, more recently, *C. auris*. These proteins also function as opsonins, activating the complement cascade, augmenting fungal internalization, and modulating the innate immune response, thereby promoting fungal clearance *in vivo* (29–32).

In this study, we investigated additional and complement-independent antifungal mechanisms of the antibody-like Lectin-Fc(IgG2a) proteins by comprehensively evaluating their structural and metabolic effects on *C. albicans*. Our findings revealed that Lectin-Fc(IgG2a) treatments exert direct microbicidal effects, influencing fungal fitness and virulence by altering cell wall polysaccharide composition, depleting intracellular micronutrient pools and lipid reserves, inducing oxidative stress, and affecting yeast protein expression and EV release. As a consequence of these pleiotropic effects, *C. albicans* yeasts become more susceptible to recognition and subsequent elimination by dendritic cells (DCs), and potentially enhancing their susceptibility to conventional antifungals. Notably, Lectin-Fc(IgG2a) proteins were more effective against strains deficient in secreted aspartyl-proteases (Saps). These findings reveal novel mechanisms of action for Lectin-Fc(IgG2a) proteins, reinforcing their potential as efficient antifungal agents and promising therapeutic options.

## MATERIALS AND METHODS

### Organisms and growth conditions

*C. albicans* SC5314 (ATCC MYA-2876) was cultured under standardized growth conditions as described (29, 31). Briefly, *C. albicans* yeasts were grown in Sabouraud broth at 37°C for 48 h under 150 rpm shaking (29, 31). Yeasts were harvested by centrifugation at 1,100 × *g* for 10 min and washed three times (3×) by resuspension/centrifugation with phosphate-buffered saline (PBS; 137 mM NaCl, 2.7 mM KCl, 1.5 mM KH_2_PO_4_, 8.1 mM Na_2_HPO_4_, pH 7.4). Yeasts were enumerated using a hemocytometer and used in subsequent experiments.

### Lectin-Fc(IgG2a) fusion protein expression and purification

Dectin-1-Fc(IgG2a) and WGA-Fc(IgG2a) were constructed and expressed as previously detailed by our group (30, 31). CHO-k1 transfectant cells were cultured in Ham’s F-12 (HyClone-GE Healthcare, MA, USA) supplemented with 10% (vol/vol) fetal bovine serum (FBS) (Cultilab, SP, Brazil), 1.2 g/L NaHCO_3_, 1.5 g/L yeast extract, 1.5 g/L peptone (Kasvi, PR, Brazil), 1% (vol/vol) nonessential amino acids, 1% penicillin/streptomycin (Gibco-Life Technologies, MA, USA), and 70 µg/mL Zeocin (Thermo Scientific, MA, USA) at 37°C in 5% CO_2_. After reaching confluence, cultures were maintained for an additional 48 h. Supernatants were collected by centrifugation at 800 × *g* for 10 min, filtered through a 0.22 μm membrane (Millipore Merck, Darmstadt, Germany), and purified using a HiTrap Protein A HP column (GE Healthcare Life Sciences, Singapore). Finally, Lectin-Fc(IgG2a) concentrations and molecular quality control were assessed by enzyme-linked immunosorbent assay (ELISA) and Western blot as described (29–31).

### Fungal growth inhibition assay

The efficacy of Lectin-Fc(IgG2a) proteins in inhibiting fungal growth has been previously investigated (29–31). Treatment affected growth kinetics but did not prevent cultures from reaching maximum growth, and the minimum inhibitory concentration (MIC) of the Lectin-Fc(IgG2a) proteins was determined for each specific case [Dectin-1-Fc(IgG2a) 12.5 μg/mL and WGA-Fc(IgG2a) 12.5 μg/mL]. To assess the influence of *C. albicans* proteases on Lectin-Fc(IgG2a)-mediated fungal growth inhibition, experiments compared the growth of wild-type (WT) strains SC5314 and M134 (parental strain) with secreted aspartyl proteinases (*sap*) mutants, including single mutants M1175 and M1628 (*sap*9^−/−^ and *sap*10^−/−^, respectively) and triple mutants M1630 and M1632 (*sap*1/2/3^−/−^ and *sap*4/5/6^−/−^, respectively), generously provided by Dr. Bernhard Hube (33–35). Broth microdilution growth inhibition assays were performed according to the CLSI protocols with slight modifications (29). In 96-well plates, yeast suspensions (200 μL of a 1 × 10^3^ *C. albicans*/mL in RPMI 1640 with 2% glucose) were incubated with serial dilutions (12.5–0.2 μg/mL) of Dectin-1-Fc(IgG2a), WGA-Fc(IgG2a), mouse IgG2a isotype control (AB_2550621; Thermo Scientific), or no treatment. Plates were incubated at 37°C in a Bioscreen (LabSystems Oy), with absorbances at 600 nm recorded hourly over 4 days. Growth curves (absorbance × time) for each Lectin-Fc(IgG2a) were individually compared to a control curve without the fusion proteins or an IgG2a isotype control. Yeasts were counted with a hemocytometer at the MIC, and the number of viable yeasts was determined by plating on Sabouraud agar. The colony-forming units (CFUs) were enumerated and compared to controls.

### Stability of Lectin-Fc(IgG2a) proteins upon incubations with *C. albicans* culture supernatants

To evaluate the stability of Lectin-Fc(IgG2a) proteins against *C. albicans* secreted proteases, yeast cultures (1.0 × 10^3^ *C. albicans*/mL) were incubated in RPMI 1640 medium supplemented with 2% glucose at 37°C for 48 h. Culture supernatants were harvested and concentrated 20-fold using Amicon Ultra-0.5 Centrifugal Filter Units (Merck). Ten microliters of the concentrated supernatants was incubated with 5 μg of Dectin-1-Fc(IgG2a) or WGA-Fc(IgG2a) in RPMI medium at 37°C with shaking for 1, 12, or 24 h. As controls, undigested (negative) or trypsin-digested Lectin-Fc(IgG2a) (0.5 µg, positive) were included. After incubation, samples were subjected to a Western blot to detect the Lectin-Fc(IgG2a). Initially, the samples were diluted in non-reducing (Tris-HCl 12.5 mM pH 6.8, 4% glycerol, 0.4% SDS, 0.005% bromophenol blue) and reducing (plus 1% β-mercaptoethanol) buffers and incubated at 95°C for 5 min. Proteins were separated by SDS-PAGE and transferred to a nitrocellulose membrane (0.45 μm, Amersham Biosciences) (29–31). The membranes were blocked with 5% milk solution in 0.1% Tris-buffered saline with Tween (TBS-T; 20 mM Tris, 150 mM NaCl, 0.1% Tween 20, pH 7.5) for 1 h and probed with 1 µg/mL goat anti-mouse IgG conjugated with alkaline-phosphatase (AP) in blocking buffer, at room temperature (RT) for 1 h. The membranes were washed 3× with TBS-T for 10 min after each incubation and finally developed using the nitro blue tetrazolium (NBT)/5-bromo-4-chloro-3-indolyl-phosphate (BCIP) (Thermo Scientific, Waltham, MA, USA) until the visualization of bands. Then, membranes were extensively washed with distilled water and imaged.

### Evaluation of the effects of Lectin-Fc(IgG2a) proteins on the levels of fungal cell wall chitin, β-glucan, and mannosylated compounds

To determine whether the Lectin-Fc(IgG2a) proteins affect the structural composition of fungal cell walls, flow cytometry and immunofluorescence microscopy were employed to evaluate the levels and accessibility of chitin, β-1,3-glucan, and mannosylated components in *C. albicans*. Sub-inhibitory concentrations (10 μg/mL) of Dectin-1-Fc(IgG2a) and WGA-Fc(IgG2a) were used as previously defined (29, 31). Yeasts (1 × 10^3^ *C. albicans*/well) were cultured in 96-well plates with either the fusion proteins or RPMI medium alone (control) at 37°C for 48 h. After incubation, yeasts were washed twice with PBS, fixed in 4% paraformaldehyde (PF), and additionally washed 3× with Dulbecco’s PBS (DPBS; PBS plus, 0.49 mM MgCl_2_ and 0.90 mM CaCl_2_, pH 7.4). To assess total chitin, yeasts were stained with 5 mg/mL Uvitex 2B in PBS at RT for 30 min. For total β-1,3-glucan, 0.1% aniline blue staining was performed under the same conditions (36, 37). Labeled yeasts were washed with PBS and visualized using an Axio Imager Microscope (Carl Zeiss MicroImaging, Inc.) with a 100× objective. Images were analyzed using ImageJ (NIH, Bethesda, MD) and Adobe Photoshop 23.5.0 (Adobe, United States). Remaining cells were analyzed by flow cytometry using a BD LSRFortessa. Fluorescence intensity was quantified using FlowJo software (v. 10.2) (Becton Dickinson, NJ, USA).

To determine the accessibility of cell wall components, yeasts were blocked in 1% BSA in PBS at RT for 1 h, in a tube revolver rotator (ThermoFisher Scientific). Yeasts were washed 3× with PBS, and each treatment was incubated with 5 μg/mL of Dectin-1-Fc(IgG2b) (β-1,3-glucan accessibility), 1 μg/mL WGA-TRICT (chitin accessibility), or 20 μg/mL of ConA-TRITC (detection of mannosylated component) in 1% BSA in DPBS for 1 h at RT. After washing, yeasts were incubated with 5 µg/mL of a goat anti-mouse IgG2b-Alexa 488 conjugate (Life Technologies) at RT for 1 h. Upon washing with PBS, yeasts were analyzed by fluorescence microscopy and flow cytometry (FL1^+^ or FL2^+^ intensity).

### Effects of Lectin-Fc(IgG2a) proteins on the fungal cell micronutrient homeostasis, oxidative stress, lipid reserves, and viability

To investigate whether Lectin-Fc(IgG2a) fusion proteins disrupt key intracellular functions, we evaluated their impact on micronutrients such as calcium (Ca^2+^), ferric iron (Fe^3+^), and zinc (Zn^2+^), lipid reserves, oxidative stress/reactive oxygen species (ROS), and viability were assessed by flow cytometry. Yeasts were co-incubated with Dectin-1-Fc(IgG2a) and WGA-Fc(IgG2a) as described. Following incubations, yeasts were harvested and washed twice with PBS. To detect intracellular Fe^3+^ pools, yeasts were incubated with 0.5 mg/mL Calcein-AM + propidium iodide (PI) (Molecular Probes, ThermoFisher Scientific) at 37°C for 20 min. For intracellular Zn^2+^ and Ca^2+^ detection, yeasts were incubated with 0.1 μM Fluozin-3AM and 0.1 μM FURA-2 at 37°C for 30 min, respectively (Molecular Probes). To measure the levels of reactive oxygen species (ROS), yeasts were labeled with 0.1 μM 5-(and 6)-chloromethyl-2′,7′-dichlorodihydrofluorescein diacetate (CM-H2DCFDA) (Molecular Probes) and 5 μM CellROX-green (Life Technologies) at 37°C for 30 min. Lipid reserves were assessed by incubating the yeasts with 2 μM BODIPY-TMR (Life Technologies) at 37°C for 30 min. Fungal viability was further evaluated by incubating the yeasts with 0.2 μM FUN-1 (Thermo Fisher Scientific) in 2% D-glucose and 10 mM Na-HEPES, at 37°C for 30 min. Following incubation, cells were washed with PBS solution or a 2% D-glucose and 10 mM Na-HEPES buffer and further incubated for 15 min at RT. Fluorescence was detected using a BD LSRFortessa flow cytometer and analyzed with FlowJo Software (v. 10.2), following the manufacturer’s instructions for each probe. Specifically for FUN-1, live cells show red/green double fluorescence, whereas dead cells show only green fluorescence.

### Transmission electron microscopy

To evaluate ultrastructural alterations in *C. albicans* following treatment with Lectin-Fc(IgG2a), yeast pellets obtained as described above were fixed overnight with a solution containing 2.5% glutaraldehyde, 4% formaldehyde, and 10 mM CaCl_2_ in 0.1 M sodium cacodylate buffer, pH 7.2, at 4°C. Subsequently, the samples were post-fixed at RT for 2 h in 1% osmium tetroxide and 0.08% potassium ferrocyanide, diluted in 0.1 M sodium cacodylate buffer. Following post-fixation, cells were dehydrated using increasing concentrations of acetone (30%, 50%, 70%, 90%, 100%, 100%, and 100%) and embedded in Spurr’s resin (Ted Pella Inc., USA). Ultrathin sections (~70 nm) were obtained using a LEICA EM UC6 ultramicrotome, placed onto 300-mesh copper grids, and contrasted with uranyl acetate and lead citrate. Sections were visualized in a transmission electron microscope (FEI Tecnai Spirit) operated at 120 kV.

### Effects of Lectin-Fc(IgG2a) on *C. albicans* biofilms

To assess the impact of the Lectin-Fc(IgG2a) treatments on *C. albicans* biofilm formation, 100 μL of a 2 × 10^6^*C. albicans*/mL diluted in RPMI-1640 medium supplemented with 5% FBS (Cultilab) and 1% penicillin-streptomycin (Gibco-Life Technologies, Waltham, MA, USA), were added to each well of a 96-well plate, with a final concentration of 10 μg/mL of each Lectin-Fc(IgG2a). As negative and positive controls for biofilm formation, respectively, amphotericin B (4 μg/mL) and PBS were used. Plates were incubated at 37°C for 24 and 48 h. The biofilm biomass was assessed by staining with 1% crystal violet for 20 min (30). Plates were washed 3× with PBS and incubated for 5 min with 100% ethanol. The content of each well was transferred to a new plate, and absorbances were measured at 570 nm. The biofilm metabolic activity was assessed with a similar plate setup using the XTT assay (36).

### Isolation of *C. albicans* EVs following treatment with Lectin-Fc(IgG2a)

*C. albicans* yeasts were cultivated in RPMI 1640 supplemented with a subinhibitory concentration of 10 μg/mL of either Dectin-1-Fc(IgG2a) or WGA-Fc(IgG2a), or PBS as a control, at 37°C for 48 h. Subsequently, the yeasts were centrifuged at 1,100 *× g* for 10 min at 4°C. Supernatants were processed further for EV isolation according to established protocols (17, 19). The supernatants were first filtered through a 0.8 μm membrane, supplemented with 1% penicillin-streptomycin, and stored at 4°C. For EV concentration, supernatants were subjected to ultrafiltration (20-fold) using a 100 kDa molecular-weight cutoff membrane, followed by ultracentrifugation at 100,000 × *g* at 4°C for 1 h. The resulting pellets were resuspended in 20 mL sterile PBS and subjected to another ultracentrifugation step. Finally, the purified EVs were resuspended in 200 μL sterile PBS, and protein concentrations were determined using the bicinchoninic acid (BCA) assay with BSA as a standard (Thermo Fisher). Sterol concentrations were determined using the Amplex Red Cholesterol Assay Kit (Thermo Fisher). Samples were preserved at -80°C until use.

### Proteomic analysis of *C. albicans* yeasts upon treatment with Lectin-Fc(IgG2a)

To analyze changes in the yeast proteome induced by Lectin-Fc(IgG2a) exposure, *C. albicans* pellets were resuspended in lysis buffer containing 7 M urea, 2 M thiourea, and 100 mM HEPES. Dithiothreitol was added to achieve a final concentration of 10 mM, and samples were incubated at 30°C for 1 h. Alkylation was performed by adding iodoacetamide to a 40 mM final concentration and incubating in the dark for 30 min. Samples were diluted with 175 μL ultrapure water (TEDIA) and digested overnight at 37°C with sequencing-grade trypsin (0.1 μg/μL in 0.1 M acetic acid, to achieve a final concentration of 16 ng/μL). Digestion was stopped by adding 10% trifluoroacetic acid (TFA) to a final concentration of 0.1%. Peptides were desalted using UltraMicroSpin columns (Harvard Apparatus) and dried in Speed Vac (Thermo Scientific). The tryptic peptides were resuspended in 10 μL of 0.1% TFA, and approximately 4 μL was injected into an in‐house packed column (15 cm × 75 µm) filled with 3 μm ReproSil C18 resin (Dr. Maisch GmbH) coupled to a Nano LC-Ultra system (Eksigent Technologies). Mass spectra were acquired in positive mode using data-dependent acquisition (DDA) with an Orbitrap analyzer (Thermo Scientific). MS1 scans covered a 350–1,800 *m*/*z* range at 60,000 resolution (at 400 *m*/*z*), with a 10,000 minimal signal and an isolation width of 2.0 *m*/*z*. The most abundant precursor ions were fragmented by collision-induced dissociation at 30% collision energy, with a dynamic exclusion set to 30 s. The acquired spectra were processed using MaxQuant 2.4.4.0 software (Max Planck Institute of Biochemistry) and searched against the *C. albicans* (strain SC5314/ATCC MYA-2876) UniProt database (June 2024) for peptide identification (38). Carbamidomethylation (MW = 57.02 Da) of cysteine residues was set by default as a fixed modification, while methionine oxidation (MW = 15.99 Da) and N-terminal acetylation as variable modifications. Two maximum missed tryptic cleavages were allowed, along with one non‐specific cleavage. Label-free quantification was enabled (multiplicity parameter set to 1). The maximum error tolerance (ppm) was defined as 20 for the first search and 6 for the main search. For protein identification and quantification, FDR was set to 0.01, with razor + unique peptides set to 1 and a minimum ratio count of 2. Gene ontology annotation on identified proteins was performed with the DAVID functional annotation tool (DAVID Bioinformatics Resources, NIAID/NIH). Protein interaction networks were mapped using the String database (https://string-db.org/). The mass spectrometry proteomics data have been deposited to the ProteomeXchange Consortium PRIDE (accession number PXD067198) (39).

### Influence of the *C. albicans* opsonization with Lectin-Fc(IgG2a) proteins on the function of bone marrow-derived dendritic cells

#### BMDC immune activation

Bone-marrow-derived dendritic cells (BMDCs) were generated from BALB/c mice, following the outlined procedures (40, 41). Male mice (8–12 weeks old) were euthanized by intraperitoneal injection of ketamine (100 mg/kg) and xylazine (10 mg/kg). Femurs and tibias were removed, and bone marrow was flushed into petri dishes containing 10 mL RPMI 1640 medium, supplemented with 10% FBS (Gibco), 1% penicillin-streptomycin (Thermo Scientific), 1% pyruvate, 1% L-glutamine, and 20 ng/mL recombinant granulocyte-macrophage colony-stimulating factor (rGM-CSF; Peprotec). Cells were cultured at 37°C/5% CO_2_ for 7 days. On day 3, an additional 10 mL of supplemented fresh medium was added. On day 7, BMDCs were harvested, counted, and seeded into 12-well plates at 2.0 × 10^6^ DCs/well, and incubated overnight at 37°C/5% CO_2_. *C. albicans* yeasts were pre-incubated for 1 h at RT with 5 µg/mL of Dectin-1-Fc(IgG2a), WGA-Fc(IgG2a), or PBS, and then washed 3× with PBS. Before infection, DCs were washed with plain RPMI medium, and *C. albicans* yeasts were added at a multiplicity of infection (MOI) of 2:1 (yeasts: BMDC). Plates were incubated for 2 h at 37°C/5% CO_2_, and wells were washed 3× with PBS to remove non-phagocytosed yeasts, and 200 µL of fresh supplemented RPMI 1640 medium was added. Cells were incubated for an additional 18 h. The supernatants were collected for future analysis, and 1 mL of cold PBS was immediately added to the plates. For immunophenotyping, the plates were kept on ice, and BMDCs were gently detached by pipetting up and down. After centrifugation (200 × *g* for 5 min), BDMCs were blocked with PBS containing 10% FBS and 2% normal mouse serum for 1 h on ice. After, cells were washed twice and incubated for 1 h with 1:200 dilutions of fluorescence-labeled antibodies: CD11c-APC (cat. no. 117310), CD40-FITC (cat. no. 102905), and MHCII-PE (cat. no. 107607) following the manufacturer’s instructions (BioLegend). After washing twice, fluorescence was measured by flow cytometry (BD LSRFortessa) and analyzed in FlowJo (v. 10.2). BMDCs were gated as CD11c+/MHC-II high population, and CD40 expression was quantified.

### Cytokine measurement by ELISA

The concentrations of TNF-α, IL-6, and IL-10 were determined in collected supernatants using ELISA following the manufacturer’s instructions (BD Biosciences). These cytokines were preferentially selected due to their central roles in antifungal immunity and dendritic cell activation pathways.

Briefly, 96-well Nunc-ImmunoTM polystyrene Maxisorp ELISA flat-bottom plates (ThermoFisher Scientific) were coated overnight at 4°C with each specific capture antibody diluted in coating buffer. Plates were washed 3× with PBS and blocked with assay diluent at RT for 1 h. After washing, 100 µL of either cytokine standard or collected supernatants was added in triplicate and incubated at RT for 2 h. Wells were washed, and biotinylated detection antibodies were added for 1 h, followed by incubation with horseradish peroxidase-conjugated streptavidin for 30 min. Plates were washed 7× and incubated with substrate solution in the dark for 30 min at RT. The reaction was stopped with 1 N sulfuric acid, and absorbances were measured at 450 with wavelength corrections at 570 nm using a Biotek ELx808TM microplate reader.

### Fungus-BMDC interactions

The interactions between fungi and BMDCs were assessed by flow cytometry as described previously (30, 31, 42). BMDCs (2.0 × 10^6^) were diluted in RPMI medium and added to each well of a 12-well plate overnight at 37°C/5% CO_2_. *C. albicans* yeasts were washed 3× with PBS and labeled with NHS-rhodamine (Thermo Scientific) at 40 µg/mL for 1 h at RT in the dark. Labeled yeasts were washed 5× and incubated for 1 h at RT with either Lectin-Fc(IgG2a) fusion proteins or PBS. After washing, yeasts were diluted in supplemented RPMI 1640 medium and added to BMDCs at an MOI of 2:1 and incubated for 1 h at 37°C/5% CO_2_. Wells were washed 3× with PBS to remove non-phagocytosed yeast, 1 mL cold PBS was added, and BMDCs were gently detached by pipetting up and down. The proportion of BMDCs associated with fluorescent yeasts was quantified by flow cytometry.

### Growth inhibition assay

The capacity of DCs to inhibit *C. albicans* growth was assessed using a CFU assay (29–31). BMDCs (2.0 × 10^5^) were seeded into 96-well plates and incubated overnight. Yeasts were pre-incubated for 1 h at RT with either Lectin-Fc(IgG2a) or PBS and washed 3×. BMDCs were washed with RPMI 1640, yeasts were added at an MOI of 2:1, and plates were incubated for 2 h at 37°C/5% CO_2_. Non-phagocyted yeasts were removed by washing 3× with PBS. Then, 200 µL of supplemented RPMI 1640 medium was added to each well, and plates were further incubated for 18 h at 37°C/5% CO_2_. BMDCs were lysed with sterile Milli-Q water, and *C. albicans* yeasts were plated on Sabouraud agar plates. CFUs were enumerated after incubation for 48 h at 37°C, and the absolute numbers of CFUs were compared among the different groups.

### Statistical analysis

All analyses were performed using the GraphPad Prism version 9.00 for Windows (GraphPad Software, San Diego, CA, USA). Differences among groups were assessed using One-way ANOVA, with a 95% confidence interval, and *P* ≤ 0.05 was considered statistically significant. Individual comparisons between groups or specifically to controls were made using the Tukey and Dunnett post-tests, respectively.

## RESULTS

### Impact of Lectin-Fc(IgG2a) treatment on *C. albicans* viability

**Fig 1:**
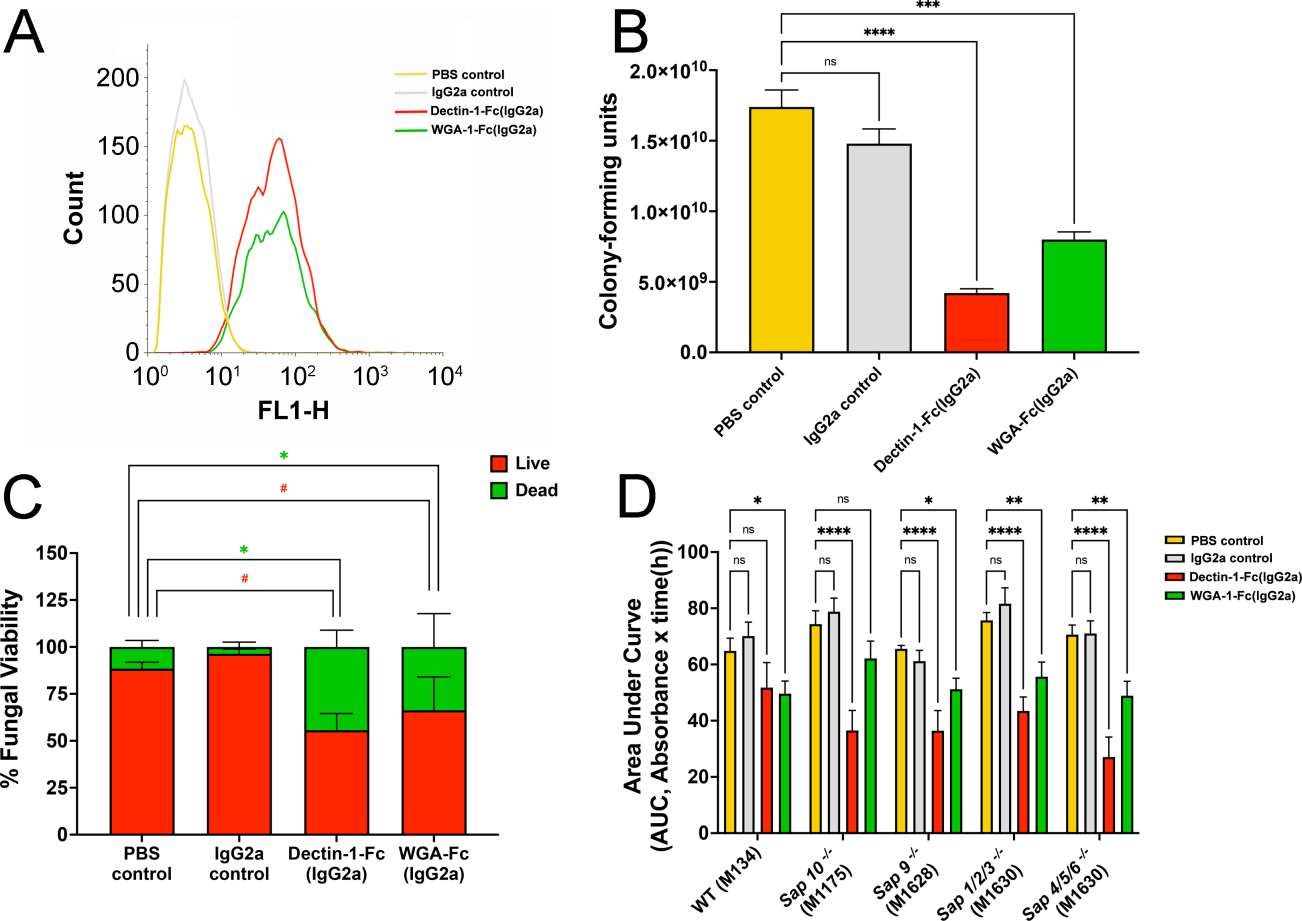
Impact of Lectin-Fc(IgG2a) on the growth and viability of *C. albicans*. (**A**) Representative fluorescence histogram of Lectin-Fc(IgG2a) [Dectin-1-Fc(IgG2a and WGA-Fc(IgG2a)] binding to *C. albicans*. (**B**) Dectin-Fc(IgG2a) and WGA-Fc(IgG2a) treatments significantly reduced CFUs after 48 h (*P* < 0.0001 and *P* = 0.0004, respectively). (**C**) Fungal viability using FUN-1 labeling indicated the proportions of live (red) and dead (green) populations after 48-h treatment with either Lectin-Fc(IgG2a). (**D**) Area under the curve of WT (M134) and single (*sap*9^−/−^ and *sap*10^−/−^) and triple (*sap*1/2/3^−/−^ and *sap*4/5/6^−/−^) SAP mutants treated with Lectin-Fc(IgG2a). An isotype IgG2a control demonstrated no binding and no impact on yeast growth. Symbols: *P ≤ 0.05, **P ≤ 0.01, ***P ≤ 0.001, ****P ≤ 0.0001, and ns, not significant.

We first evaluated and compared the ability of both Lectin-Fc(IgG2a) [Dectin-1-Fc(IgG2a) or WGA-Fc(IgG2a)] to bind *C. albicans* yeast, cultivated in RPMI-MOPS-defined medium as recommended by CLSI guidelines (Fig. 1A). The Fc(IgG2a isotype) control demonstrated no binding to *C. albicans* yeasts. Following treatment with each Lectin-Fc(IgG2a) variant, we assessed fungal viability (Fig. 1B and C).

Co-incubations with Dectin-1-Fc(IgG2a) or WGA-Fc(IgG2a) significantly reduced the number of CFUs, indicating a marked inhibitory effect on yeast growth (*P* < 0.0001 and *P* = 0.0004, respectively; Fig. 1B). Viability staining coupled with flow cytometry further confirmed these findings, showing that treatment with Dectin-1-Fc(IgG2a) or WGA-Fc(IgG2a) decreased the proportion of live yeasts by approximately 33% and 22%, respectively, compared to untreated or Fc(IgG2a isotype) controls, which had no impact on fungal growth nor viability (Fig. 1C).

### Lectin-Fc(IgG2a) efficiently inhibits the growth of *C. albicans* protease mutants

The inhibitory potential of Lectin-Fc(IgG2a) proteins [Dectin-1-Fc(IgG2a) and WGA-Fc(IgG2a)] on the growth of different *C. albicans* strains was comprehensively examined (Fig. 1D). Dectin-1-Fc(IgG2a) significantly impaired the growth of the WT parental strain (M134, Fig. 1D). However, a more pronounced inhibitory effect was observed on the growth of secreted aspartyl proteases (sap)-deficient mutants, including *sap*1/2/3^−/−^, *sap*4/5/6^−/−^, *sap*9^−/−^, and *sap*10^−/−^ mutants. WGA-Fc(IgG2a) also inhibited the growth of the WT strain (M134). In addition, *C. albicans sap*9^−/−^, *sap*1/2/3^−/−^, and the *sap*4/5/6^−/−^ mutants were markedly more susceptible to growth inhibition. Overall, treatment with the Lectin-Fc(IgG2a) fusion proteins resulted in a significantly impaired *C. albicans* growth, with the strongest effect observed in the *sap*4/5/6^−/−^ mutant strain.

### *C. albicans* protease activity against Lectin-Fc(IgG2a)

Given the heightened susceptibility of *sap*-deficient *C. albicans* strains to growth inhibition by Lectin-Fc(IgG2a) fusion proteins, we investigated whether these proteins could be potentially hydrolyzed by fungal secreted proteases. Culture supernatants from *C. albicans* grown for 48 h were incubated with 5 µg of Lectin-Fc(IgG2a) for 1, 12, and 24 h, and the presence of intact Lectin-Fc(IgG2a) was assessed by Western blotting (Fig. S1A and B). A gradual reduction in the Dectin-1-Fc(IgG2a) levels was observed over time, with the fusion protein still detectable even after 24 h of incubation with the supernatant (1 h, *P* = 0.0002; 12 h, *P* < 0.0001; and 24 h, *P* < 0.0001; Fig. S1A and B). In comparison, more extensive digestion was detected for the WGA-Fc(IgG2a); however, this fusion remained partially intact after 24 h (1 h *P* < 0.0001, 12 h *P* < 0.0001, 24 h *P* <0.0001; Fig. S1A and B).

### Lectin-Fc(IgG2a) affected fungal protein expression

**Fig 2:**
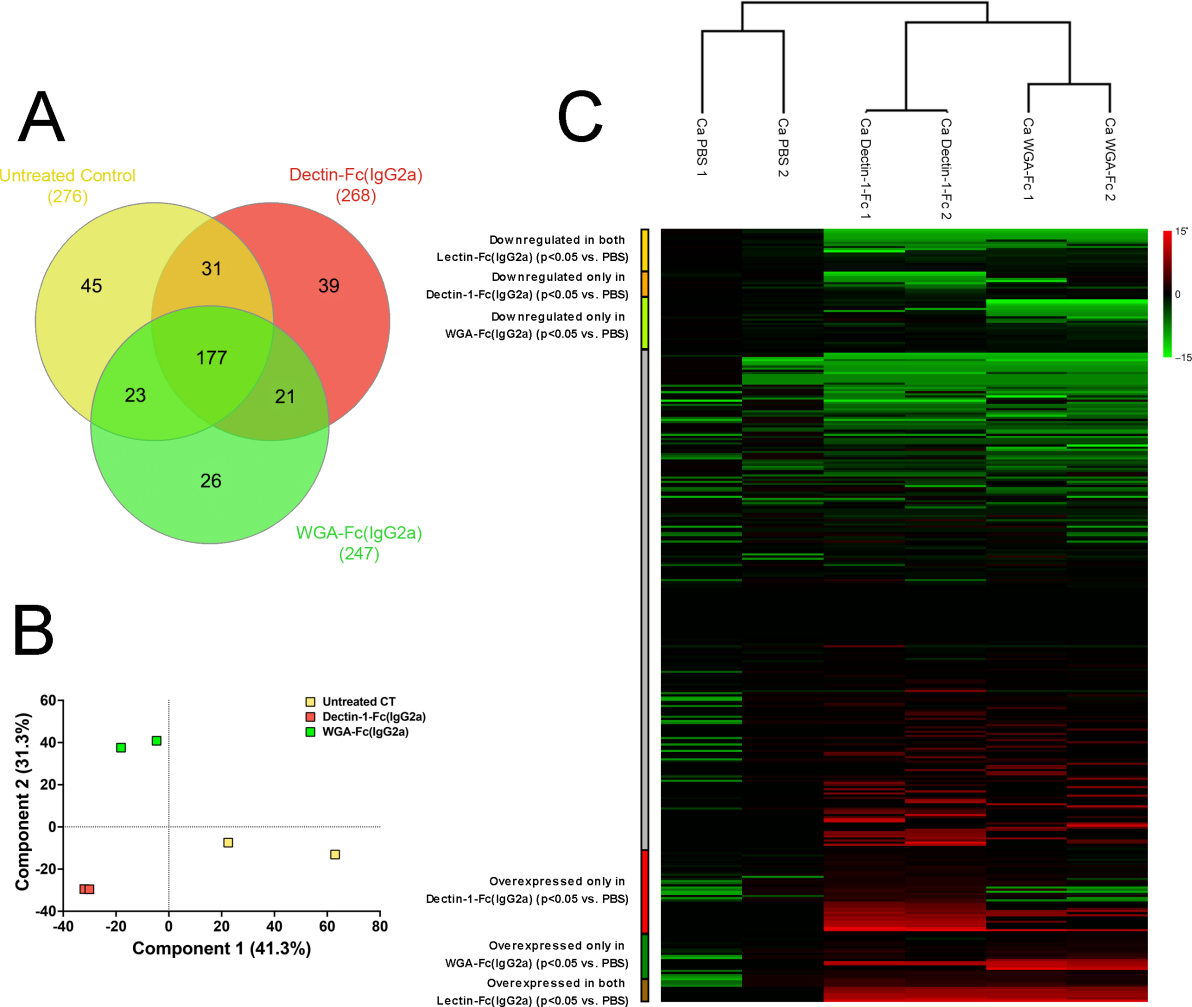
Lectin-Fc(IgG2a) treatment altered protein expression in *C. albicans* yeast. (**A**) Venn diagrams displaying the number of detected proteins in each Lectin-Fc(IgG2a) treatment compared to controls. (**B**) Principal component analysis displaying clustering of the control or Lectin-Fc(IgG2a)-treated groups. (**C**) Comprehensive heat map depicting proteins downregulated by both Lectin-Fc(IgG2a) treatments, specifically downregulated in either Dectin-1-Fc(IgG2a) or WGA-Fc(IgG2a) in contrast with upregulated proteins in both Lectin-Fc(IgG2a) in comparison to controls.

We conducted proteomics analysis to evaluate the impact of Lectin-Fc(IgG2a)s [Dectin-1-Fc(IgG2a) and WGA-Fc(IgG2a)] incubations on the protein expression of *C. albicans* yeasts. The Venn diagram (Fig. 2A) illustrates that mass spectrometry identified a total of 362 proteins across all experimental conditions, with 177 proteins commonly expressed among untreated controls and Lectin-Fc(IgG2a) treatments. Dectin-1-Fc(IgG2a) treatments led to the exclusive expression of 39 proteins, along with 31 proteins shared with the untreated control, and 21 proteins WGA-Fc(IgG2a)-treated by *C. albicans*. WGA-Fc(IgG2a) treatment resulted in 26 uniquely expressed proteins, while 23 proteins were shared with the control yeasts. Additionally, 45 proteins were exclusively expressed in untreated control yeasts (Fig. 2A). Principal component analysis (PCA) further demonstrated a clear segregation between controls and Lectin-Fc(IgG2a)-treated groups (Fig. 2B), reflecting distinct proteomic profiles and consistent clustering among replicates. This divergence was corroborated by hierarchical clustering and heat map analysis, which revealed distinct expression patterns across the conditions (Fig. 2C; Table S1).

**Fig 3:**
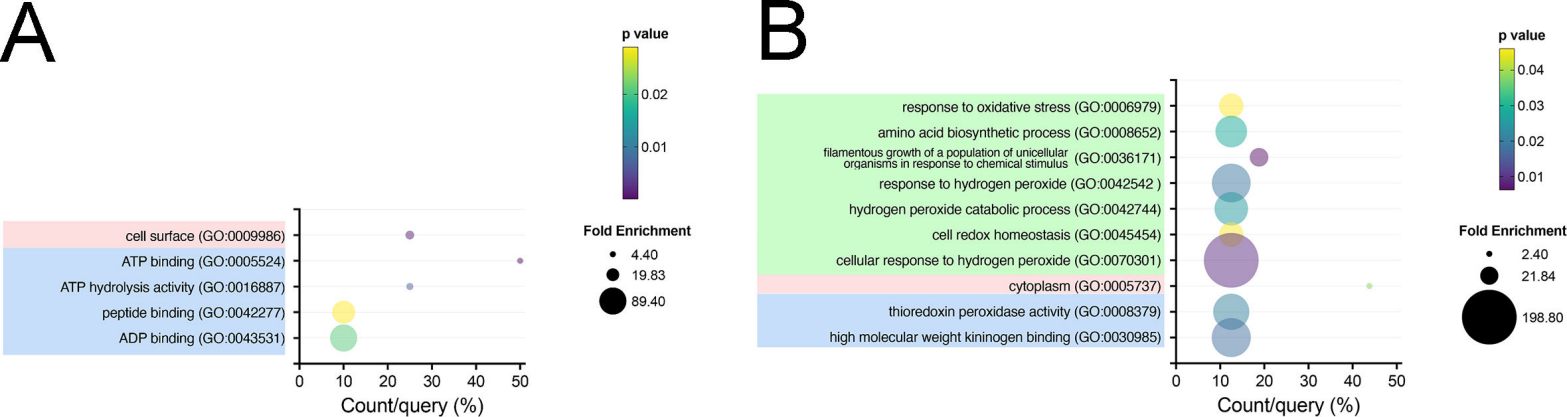
Gene ontology (GO) enrichment analysis of proteins altered by Lectin-Fc(IgG2a) treatments. (**A**) GO Enrichment analysis of commonly downregulated proteins upon treatment with either Lectin-Fc(IgG2a). (**B**) GO Enrichment analysis of commonly upregulated proteins upon treatment with either Lectin-1-Fc(IgG2a). Green rectangles: GO biological processes; pink rectangles: GO cellular components and blue rectangles: GO molecular functions.

Enrichment analyses of differentially expressed proteins comparing the untreated control and Lectin-Fc(IgG2a) [Dectin-1-Fc(IgG2a) and WGA-Fc(IgG2a)] revealed distinct protein expression profiles (Fig. 3). Treatment with either Dectin-1-Fc(IgG2a) or WGA-Fc(IgG2a) resulted in decreased expression of proteins associated with the cell surface (GO:0009986) and molecular functions including ATP binding (GO:0005524), ADP binding (GO:0043531), ATP hydrolysis activity (GO:0016887), and peptide binding (GO:0042277) (Fig. 3A, *P* < 0.05).

Conversely, treatment with both Lectin-Fc(IgG2a) enhanced the expression of proteins involved in biological processes such as response to oxidative stress (GO:0006979), response to hydrogen peroxide (GO:0042542), hydrogen peroxide catabolic process (GO:0042744), cell redox homeostasis (GO:0045454), and cellular response to hydrogen peroxide (GO:0070301). Additional enriched processes included amino acid biosynthetic process (GO:0008652) and filamentous growth of a population of unicellular organisms in response to chemical stimulus (GO:0036171). Relevant cellular components and molecular functions include cytoplasm (GO:0005737) and thioredoxin peroxidase activity (GO:0008379) (Fig. 3B; *P* < 0.05). Comprehensive protein interaction networks are presented for commonly downregulated (Fig. S2A) and upregulated proteins (Fig. S2B). Additionally, KEGG pathway enrichment analysis on differentially regulated proteins revealed that co-incubations with Lectin-Fc(IgG2a) downregulated proteins participating in glycolysis/gluconeogenesis (path:cal00010), while proteins involved in the biosynthesis of amino acids were upregulated (path: cal01230; Fig. S3).

### Lectin-Fc(IgG2a) treatments impacted the morphology of *C. albicans* yeasts

**Fig 4:**
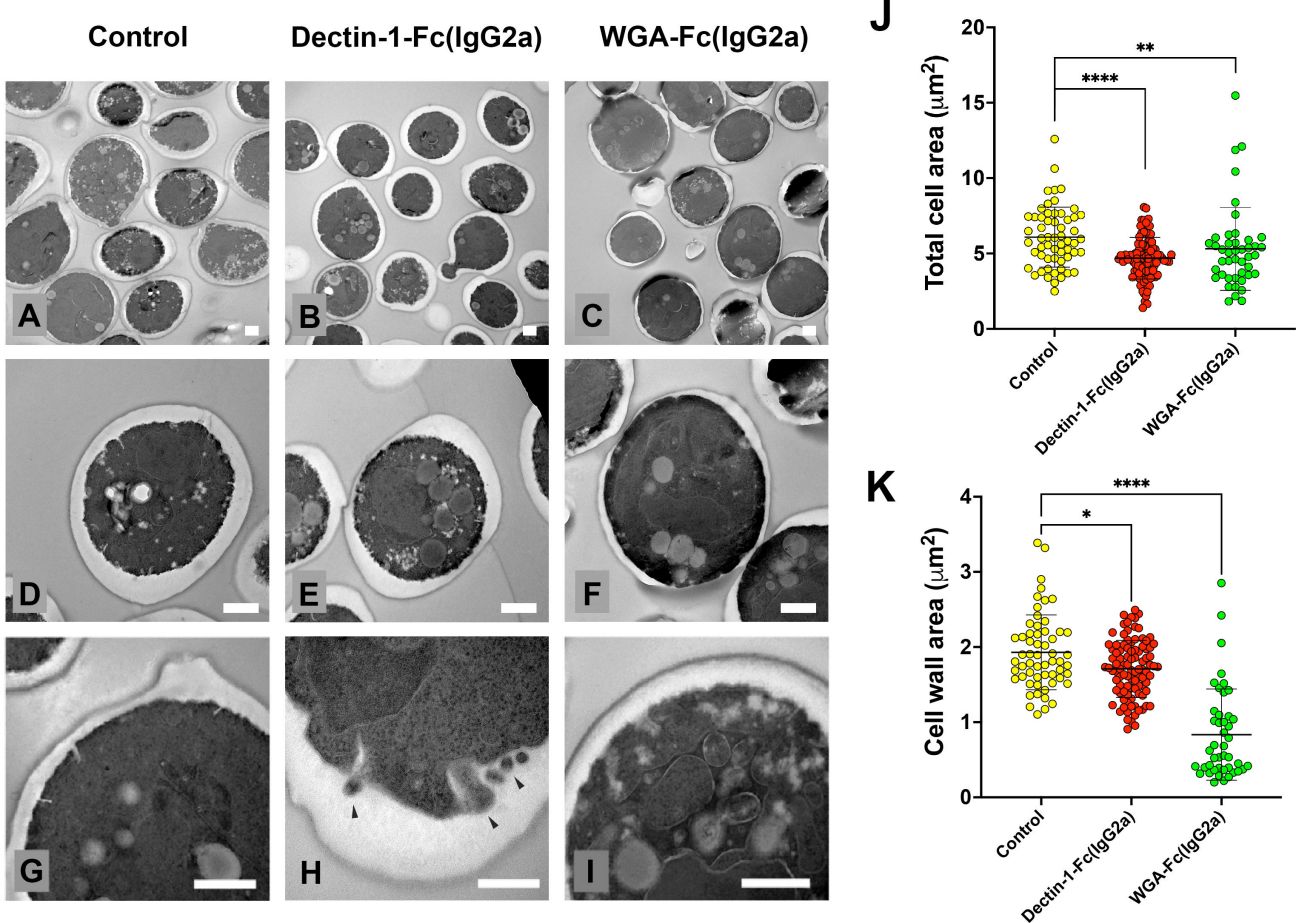
Ultrastructural alterations in *C. albicans* SC5314 after Lectin-Fc(IgG2a) treatment. (**A, D, and G**) Control yeasts exhibited an intact ultrastructure with a compact cell wall, a continuous cytoplasmic membrane, and an electron-dense cytoplasm. (**B, E, and H**) In contrast, yeasts treated with Dectin-1-Fc(IgG2a) showed increased vacuolization (**B and E**) and vesicle accumulation (black arrows in panel H). (**C, F, and I**) WGA-Fc(IgG2a)-treated cells also displayed compromised cytoplasm integrity and intense vacuolization. The scale bars were set at 500 nm. (**J**) Treatment with either Lectin-Fc(IgG2a) resulted in a significant reduction in cellular dimensions (23%, *P* < 0.0001 and 13%, *P* = 0.005, respectively). (**K**) Treatment with either Lectin-1-Fc(IgG2a) resulted in a thinner cell wall in comparison to controls (11%, *P* = 0.044 and 57%, *P* < 0.0001 reduction, respectively). Symbols: *P ≤ 0.05, **P ≤ 0.01, and ****P ≤ 0.0001.

Transmission electron microscopy revealed substantial morphological alterations in *C. albicans* yeasts following Lectin-Fc(IgG2a) treatments. Control yeasts exhibited an intact cellular ultrastructure characterized by loosely organized cell walls (Fig. 4A), along with a more homogeneous cytoplasm (Fig. 4D), and fewer visible internal structures (Fig. 4G). In contrast, treatment with Dectin-1-Fc(IgG2a) or WGA-Fc(IgG2a) significantly reduced cellular dimensions by 23% (*P* < 0.0001) and 13% (*P* = 0.005), respectively (Fig. 4B, C, and J). Treatments with either Lectin-Fc(IgG2a) also resulted in a more compact cell wall (Fig. 4C and J), with the WGA-Fc(IgG2a) demonstrating a more notable reduction compared to Dectin-1-Fc(IgG2a) (57%, *P* < 0.0001 and 11%, *P* = 0.044 reduction; Fig. 4E and F, respectively).

Additionally, treatments with Lectin-Fc(IgG2a) also led to a more heterogeneous and disorganized cytoplasm, accompanied by an increased number of large electron-lucent membranous structures (Fig. 4E, F, H, and I). Dectin-1-Fc(IgG2a) induced apparent accumulation of EVs within the periplasmic space (Figure H). Furthermore, yeasts treated with WGA-Fc(IgG2a) exhibited compromised organelle organization, characterized by fewer electron-dense regions and the formation of large intracellular multivesicular bodies (Fig. 4C, F, and I).

### Lectin-Fc(IgG2a) affects the composition of cell wall carbohydrates, including chitin, β-glucan, and mannosylated compounds in *C. albicans*

Proteomic data revealed that treatment with Lectin-Fc(IgG2a) downregulated the expression of several cell wall-associated proteins, including heat-shock protein 70 (Hsp70; P41797, SSA1), phosphoglycerate kinase (P46273, PGK1), AAA family ATPase (Q59WG3, CDC48), plasma membrane ATPase (PMA1, A0A1D8PJ01), and F1F0 ATP synthase (ATP2, A0A1D8PKZ9) (Table S1). Additionally, both Lectin-Fc(IgG2a) reduced the levels of ATP-dependent 6-phosphofructokinase (Q5AGZ8, PFK2) and phosphoglycerate kinase (P46273, PGK1) both mapped to the glycolysis/gluconeogenesis pathway (GO:0006096/ GO:0006094).

**Fig 5:**
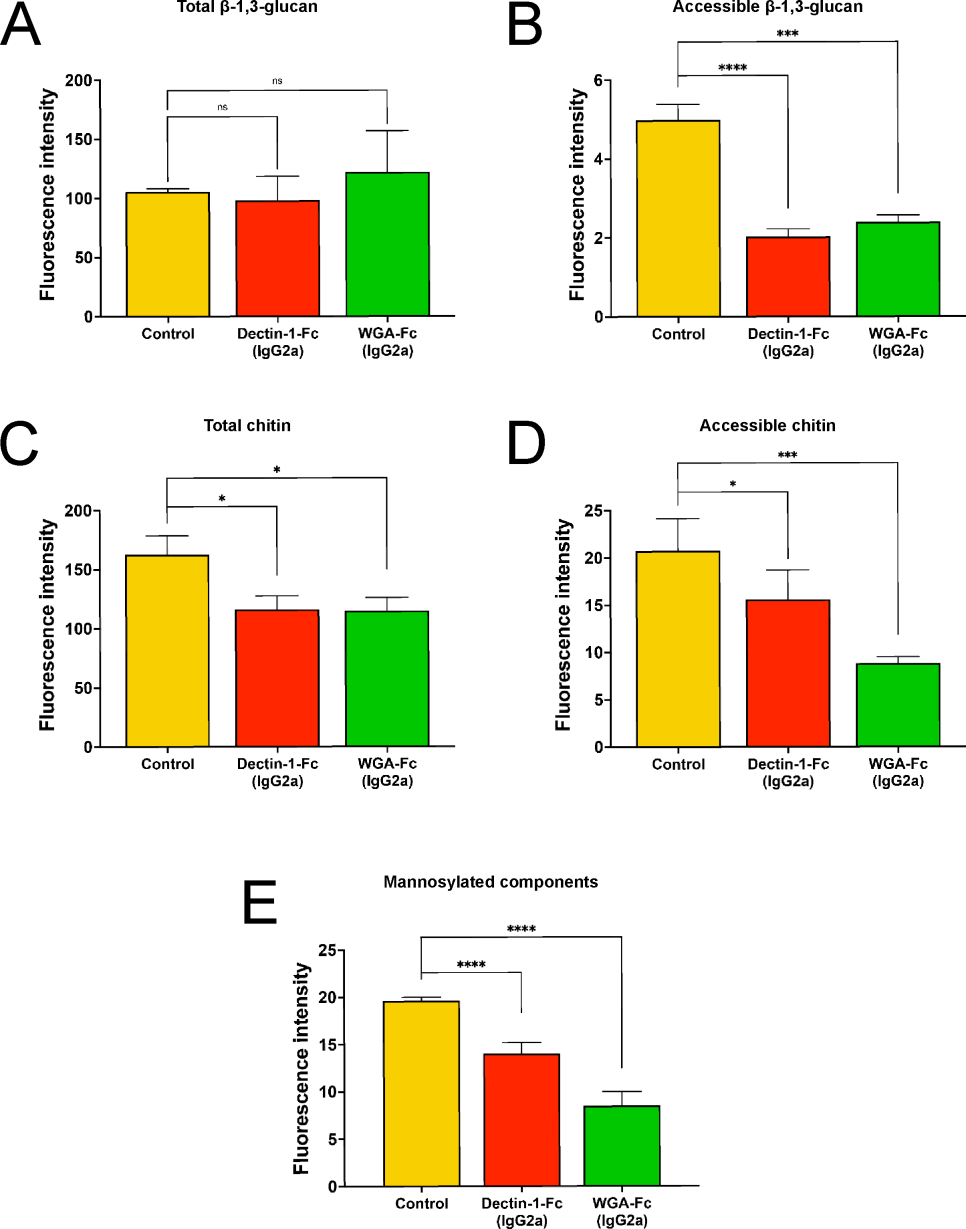
Lectin(IgG2a) treatments modified the fungal cell wall. (**A**) Treatment with Dectin-1-Fc(IgG2a) and WGA-Fc(IgG2a) resulted in no alteration of total β-1,3-glucans, as detected by aniline blue. However, both Lectin-Fc(IgG2a) treatments resulted in (**B**) decreased accessibility of β-1,3-glucans (Dectin-1-Fc(IgG2a), *P* < 0.0001 and WGA-Fc(IgG2a), *P* = 0.0004). (**C and D**) Treatment with Lectin-Fc(IgG2a) resulted in a decrease of (**C**) total (*P* = 0.032 and *P* = 0.029, respectively) and (**D**) accessible chitin content on the fungal cell wall. (**E**) Treatment with Lectin-Fc(IgG2a) overall resulted in a decrease in the levels of mannosylated components of the *C. albicans* cell wall (*P* = 0.0017 and *P* < 0.0001, respectively). Symbols: *P ≤ 0.05, ***P ≤ 0.001, ****P ≤ 0.0001, and ns, not significant.

These findings, combined with TEM ultrastructural differences, corroborate structural and composition changes in cell wall carbohydrates, including chitin, β-glucan, and mannosylated compounds. Total β-1,3-glucan, as detected with soluble aniline blue, remained unchanged after treatment with both Lectin-Fc(IgG2a) proteins (Fig. 5A). However, β-1,3-glucan accessibility, assessed by Dectin-1-Fc(IgG2b) labeling, was significantly reduced, suggesting remodeling toward a more compact architecture compared to control yeasts (*P* < 0.0001 and *P* = 0.0004, respectively; Fig. 5B). Immunofluorescence further confirmed diminished β-1,3-glucan labeling in Dectin-1-Fc(IgG2a)-treated yeasts, which exhibited a diffuse pattern. In contrast, WGA-Fc(IgG2a) treatment showed less pronounced dotted labeling, differing from the distinct, bright gear-like pattern of untreated cells (Fig. S4A).

Similarly, total chitin content [Dectin-1-Fc(IgG2a), *P* = 0.032 and WGA-Fc(IgG2a), *P* = 0.029] (Fig. 5C) and chitin accessibility (Fig. 5D) were significantly reduced after treatment with both Lectin-Fc(IgG2a), suggesting a compromised chitin structure and accessibility, as supported by fluorescence microscopy (Fig. S4A). Moreover, Dectin-1-Fc(IgG2a) and WGA-Fc(IgG2a) decreased the labeling of mannosylated components (*P* = 0.0017 and *P* < 0.0001, respectively; Fig. 5E), corroborated by fluorescence images (Fig. S4B) and a thinner mannan layer observed by TEM. Collectively, these findings demonstrate that Lectin-Fc(IgG2a) proteins perturb the carbohydrate composition and organization of the *C. albicans* cell wall.

### Treatments with Lectin-Fc(IgG2a) led to oxidative stress and micronutrient imbalance in *C. albicans* yeasts

**Fig 6:**
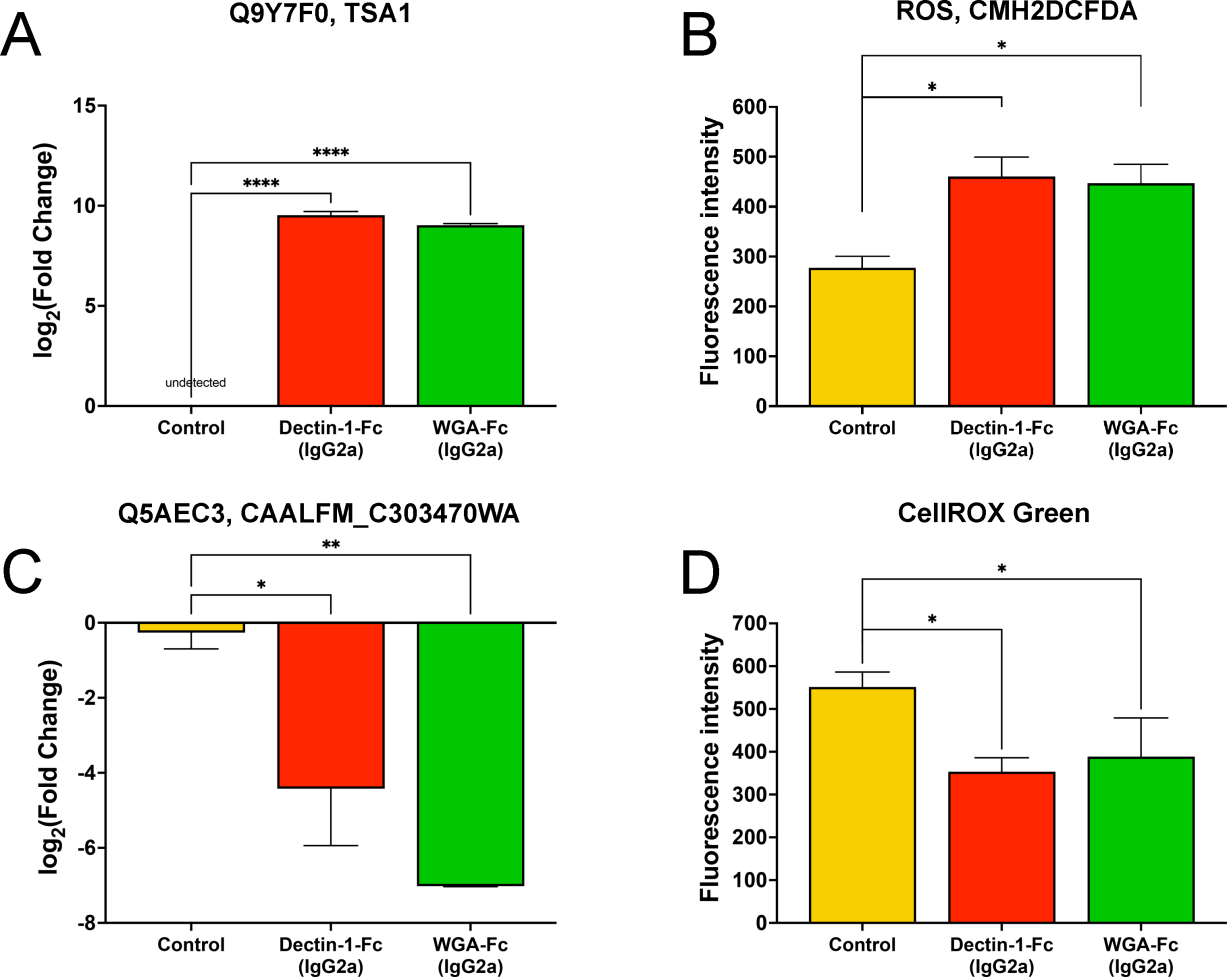
Lectin-Fc(IgG2a) treatments led to oxidative stress in *C. albicans*. (**A**) Both Lectin-Fc(IgG2a) induced high levels of expression of a peroxiredoxin (TSA1, Q9Y7F0), as a result of (**B**) elevated cytoplasmic ROS levels detected by CMH2DCFDA. (**C**) Downregulation of a succinate-semialdehyde dehydrogenase (Q5AEC3), involved in oxidative metabolism, and therefore, resulting in a reduction of (**D**) oxidative CellROX reacting species. Symbols: *P ≤ 0.05, **P ≤ 0.01, and ***P ≤ 0.001.

Proteomic data indicated that treatment with Lectin-Fc(IgG2a) induced oxidative stress in *C. albicans* (Fig. 6). Both Lectin-Fc(IgG2a) treatments increased levels of peroxiredoxin (Q9Y7F0, TSA1), a peroxidase involved in response to oxidative stress, hydrogen peroxide detoxification, and maintenance of cell redox homeostasis (Fig. 6A; Table S1). To further evaluate the oxidative stress, we performed flow cytometry using CM-H2DCFDA and CellROX-green probes. Lectin-Fc(IgG2a)-treated yeasts exhibited significantly higher levels of cytoplasmic reactive oxygen species (ROS) detected with CM-H2DCFDA compared to controls (Fig. 6B; *P* = 0.0071 for both).

Conversely, treatment resulted in decreased levels of succinate-semialdehyde dehydrogenase (Q5AEC3, CAALFM_C303470WA) (Fig. 6C), ATP synthase (A0A1D8PKZ9, ATP2), and plasma membrane ATPase (A0A1D8PJ01, PMA1), all of which are associated with oxidative phosphorylation (GO:0042776). Reduced mitochondrial activity was further supported by decreased mitochondrial ROS generation detected by CellROX-green staining in Lectin-Fc(IgG2a)-treated yeasts [Dectin-1-Fc(IgG2a), *P* = 0.015 and WGA-Fc(IgG2a), *P* = 0.05] (Fig. 6D).

**Fig 7:**
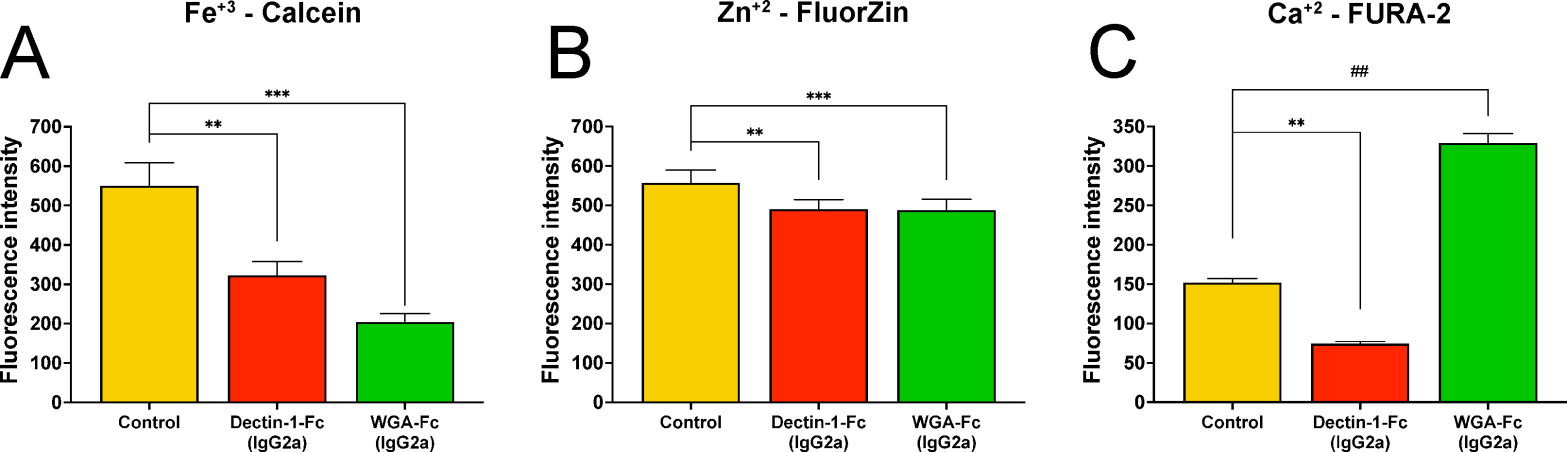
Lectin-Fc(IgG2a) treatment altered *C. albicans* intracellular micronutrient levels. Levels of essential micronutrient pools serving as cofactors for several downregulated enzymes, including numerous ADP/ATP binding and ATPases, were measured: (**A**) iron (Fe^3+^ detected with Calcein), (**B**) zinc (Zn^2+^ detected with Fluozin), and (**C**) calcium (Ca^2+^ detected with Fura-2). Overall, treatments decreased micronutrients, except for increased Ca^2+^ after WGA-Fc(IgG2a) treatment. Symbols: **P ≤ 0.01, and ***P ≤ 0.001; ^##^*P* < 0.01 [WGA-Fc(IgG2a) > control].

The differential expression of proteins involved in ADP/ATP binding, ATP hydrolysis activity, and peptide binding [AAA family ATPase, Q59WG3, CDC48; F1F0 ATP synthase subunit beta, A0A1D8PKZ9, ATP2; H(+)-exporting P2-type ATPase, A0A1D8PJ01, PMA1; Hsp70 family chaperone, P41797, HSP70 and adenyl-nucleotide exchange factor, Q96VB9, MSI3; Table S1] upon treatment with the Lectin-Fc(IgG2a), prompted us to measure relevant intracellular micronutrients that function as enzymatic co-factors (Fig. 7). Calcein-AM staining revealed significantly lower intracellular Fe^3+^ levels in yeasts treated with Dectin-1-Fc(IgG2a) and WGA-Fc(IgG2a) compared to controls (*P* = 0.0034 and *P* = 0.0002, respectively; Fig. 7A). A similar trend was observed for intracellular Zn^2+^ levels measured by Fluozin staining (*P* = 0.0071 and *P* = 0.0004, respectively; Fig. 7B). In contrast, Dectin-1-Fc(IgG2a) decreased intracellular Ca^2+^, while WGA-Fc(IgG2a) increased in the Ca^2+^ intracellular pool (*P* = 0.013, Fig. 7C).

### Treatments with the Lectin-Fc(IgG2a) impacted lipid reserves and EVs secretion in *C. albicans*

**Fig 8:**
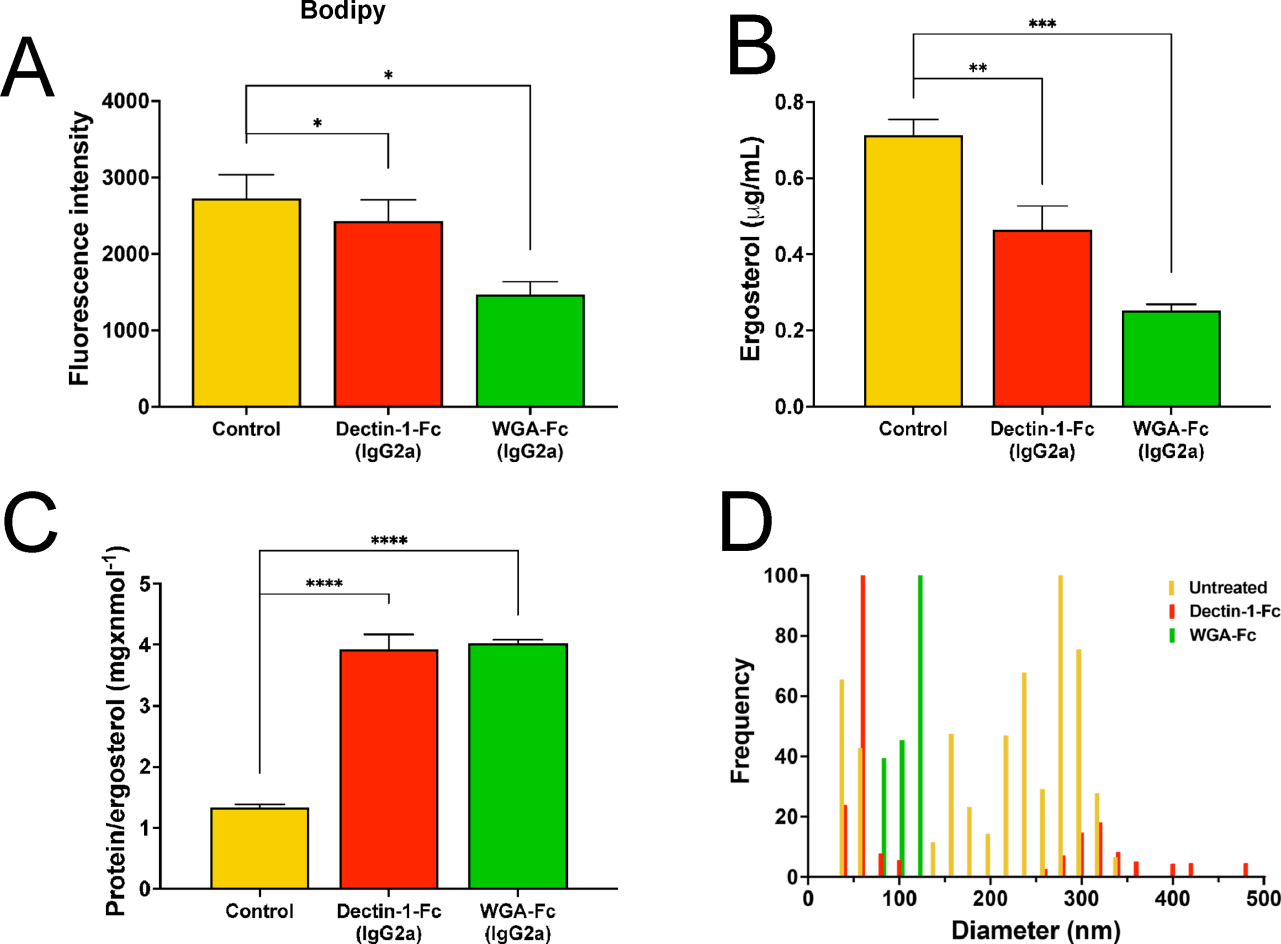
Lectin-Fc(IgG2a) treatment led to cytoplasmic disorganization, hypervacuolization, and vesicle accumulation. (**A**) Neutral lipid reserve levels (detected by BODIPY) were fairly decreased after treatment with Lectin-Fc(IgG2a). (**B**) EV secretion assessed by total sterol content was decreased. (**C**) As the protein levels in EVs were not impacted by the Lectin-Fc(IgG2a) treatment, it resulted in higher protein/ergosterol ratio, indicating that the EV cargo could be more compact in smaller membranous structures, which were further confirmed by (**D**) dynamic light scattering (DLS), displayed an overall reduction in EV size upon *C. albicans* treatment with either Lectin-Fc(IgG2a). Symbols: *P ≤ 0.05, **P ≤ 0.01, ***P ≤ 0.001, and ****P ≤ 0.0001.

TEM analysis revealed extensive cellular vacuolization and accumulation of vesicle-like structures following treatment with Lectin-Fc(IgG2a), prompting further investigation into the impact on lipid reserves and EV release. BODIPY staining showed a significant reduction in neutral lipid content in Lectin-Fc(IgG2a)-treated cells [*P* = 0.025 for Dectin-1-Fc(IgG2a) and *P* = 0.013 for WGA-Fc(IgG2a); Fig. 8A]. As expected, EV levels, measured by total ergosterol content in EVs collected from treated cells, were significantly lower, suggesting reduced EV release [Dectin-1-Fc(IgG2a), *P* = 0.0073 and WGA-Fc(IgG2a), *P* = 0.0003; Fig. 8B]. Although the overall protein concentration in EVs remained unchanged (data not shown), a significantly higher protein/ergosterol ratio (*P* < 0.0001 for both; Fig. 8C) was observed in EVs extracted from Lectin-Fc(IgG2a)-treated yeasts, suggesting altered cargo packing and reduced EV size. DLS analysis further supported the EV’s size reduction upon treatment (Fig. 8D). EVs from control cultures displayed a population of small EVs ranging from 40 to 60 nm and medium EVs from 140 to 340 nm (average diameter of 201.8 nm). In contrast, EVs recovered after Dectin-1-Fc(IgG2a) treatment consisted primarily of small EVs, ranging from 40 to 100 nm (predominantly 60 nm) and less frequent medium EVs, ranging from 260 to 360 nm (average diameter of 150 nm). Finally, EVs isolated from WGA-Fc(IgG2a)-treated cultures ranged from 80 to 120 nm (average diameter of 98.0 nm).

### Lectin-Fc(IgG2a) impacted *C. albicans* biofilm formation  

**Fig 9:**
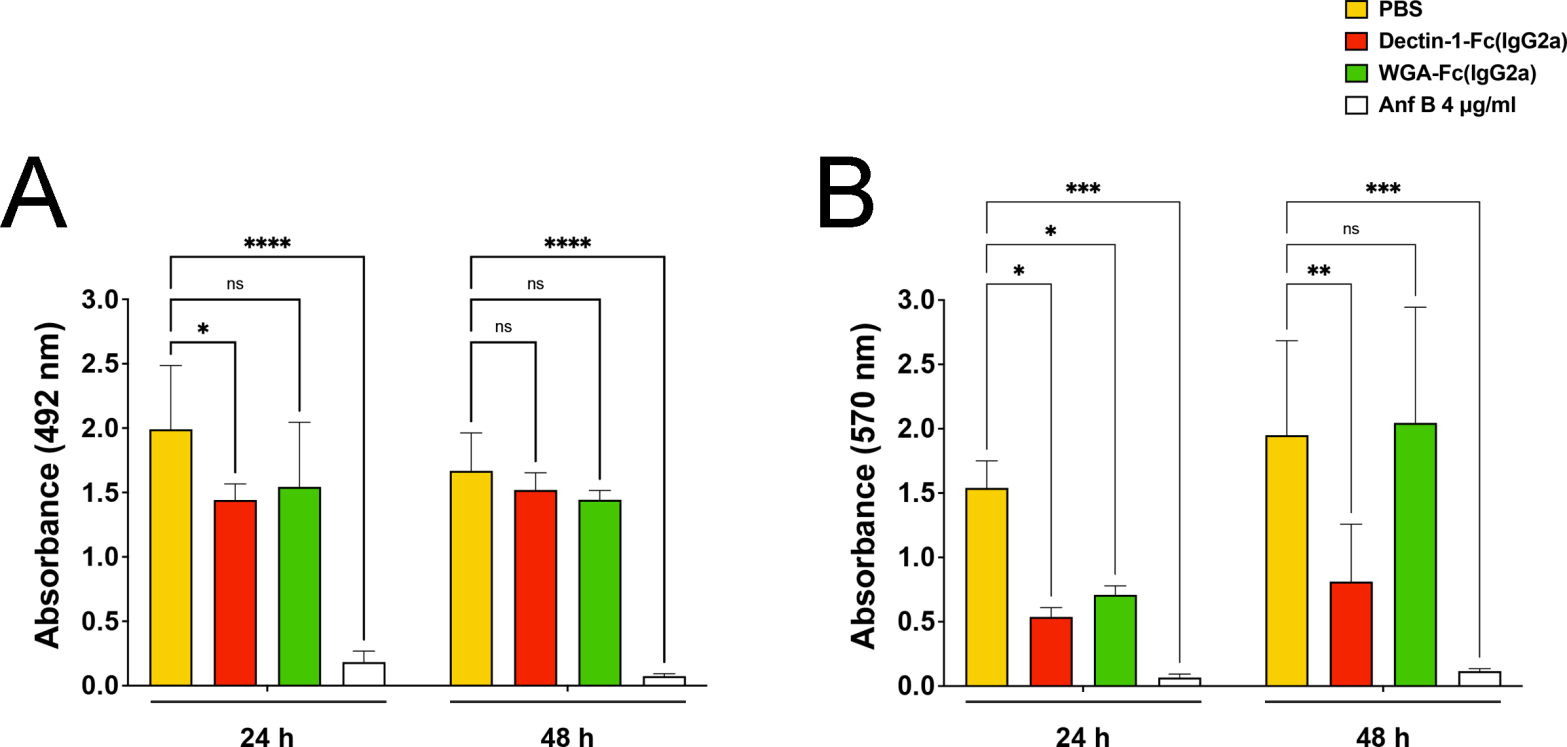
Effects of Lectin-Fc(IgG2a) on *C. albicans* biofilms. Treatment with Dectin-Fc(IgG2a) greatly impacted both (**A**) biofilm biomass and (**B**) metabolic activity of biofilms at 24 h compared to controls. In turn, WGA-Fc(IgG2a) demonstrated only an impact on the metabolic activity at 24 h of co-incubations. Amphotericin B (4 μg/mL) was used as a control for the inhibition of *C. albicans* biofilm formation. *P ≤ 0.05; **P ≤ 0.01; ***P ≤ 0.001 ; ****P ≤ 0.0001; and ns, not significant.

*C. albicans* biofilms cultivated under standard conditions were evaluated to determine the effects of Lectin-Fc(IgG2a) on biomass and metabolic activity (Fig. 9). Treatment with Dectin-1-Fc(IgG2a) significantly reduced biofilm biomass compared to the PBS control at 24 h (*P* < 0.05; Fig. 9A), while WGA-Fc(IgG2a) treatment resulted in a non-significant reduction. Regarding biofilm metabolic activity, both Lectin-Fc(IgG2a) caused a marked and significant reduction compared to PBS (*P* < 0.05; Fig. 9B) at 24 h. However, at 48 h, only the Dectin-1-Fc(IgG2a) treatment maintained a significant reduction in metabolic activity compared to untreated biofilms (*P* < 0.05; Fig. 9B).

### Lectin-Fc(IG) treatments with *C. albicans* rendered yeasts that enhanced BMDC activation

**Fig 10:**
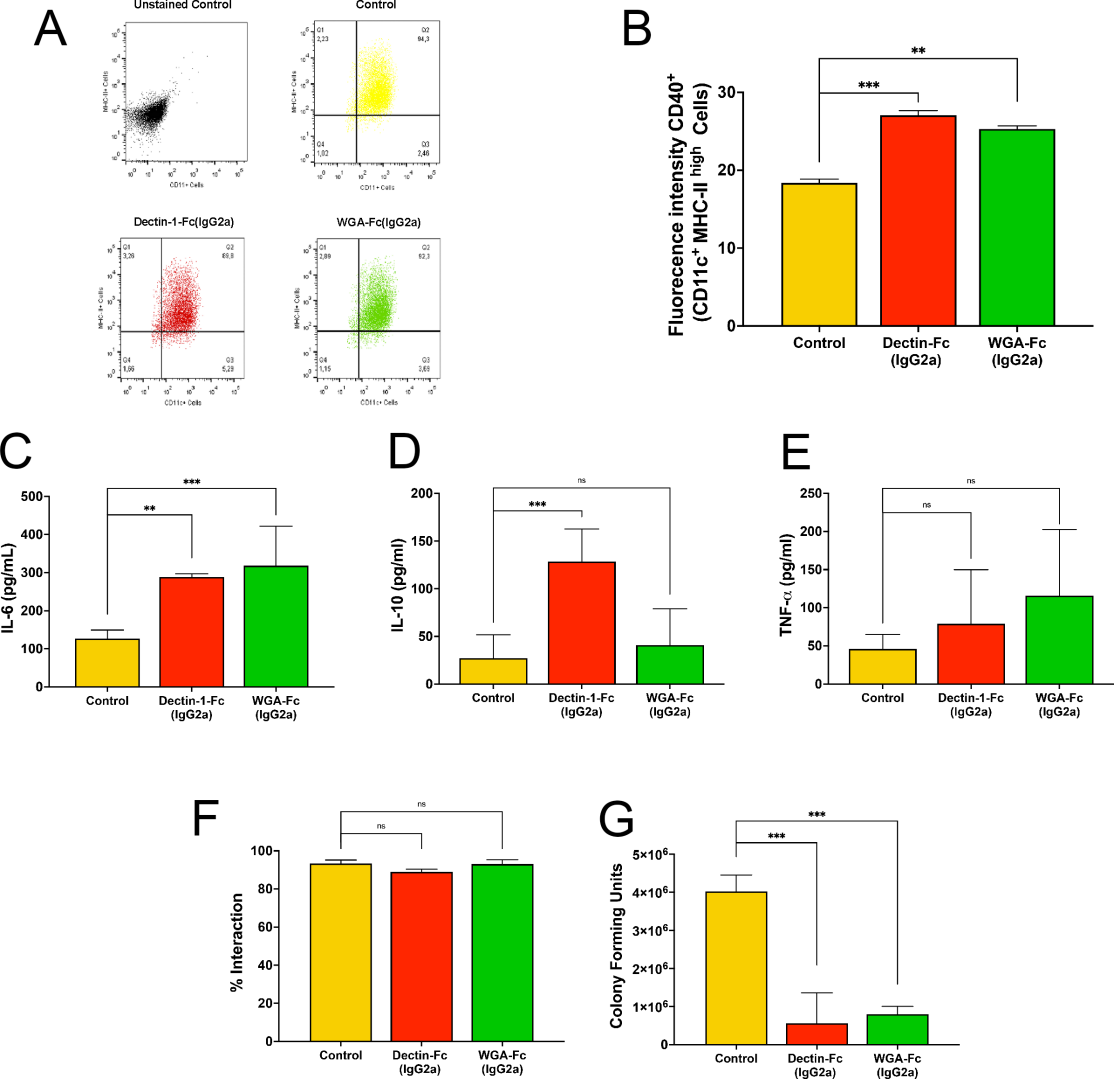
Lectin-Fc(IgG2a) enhanced BMDCs activation, cytokine production, and fungal killing. (**A**) Dot plot indicating the CD11c+/MHC-II high BMDCs population in control and after treatment with Dectin-1-Fc(IgG2a) and WGA-Fc(IgG2a). (**B**) CD40 expression in BMDCs CD11c+/MHC-II high cells after exposure to opsonized yeasts. (**C and E**) Cytokine quantification by ELISA of (**C**) IL-6, (**D**) IL-10, and (**E**) TNF-α produced by BMDCs after infection with Lectin-Fc(IgG2a)-opsonized *C. albicans* yeast. (**F**) Interaction rates of Lectin-Fc(IgG2a)-opsonized *C. albicans* with BMDCs and (**G**) killing assay demonstrating the enhancement of the antifungal activity of BMDC and a lower number of recovered CFUs after Lectin-Fc(IgG2a)-opsonization. **P ≤ 0.01; ***P ≤ 0.001; and ns, not significant.

To assess the immunomodulatory effects of Lectin-Fc(IgG2a), BMDCs were infected with *C. albicans* yeasts pre-incubated with Dectin-1-Fc(IgG2a) or WGA-Fc(IgG2a), and functional responses were evaluated (Fig. 10). BMDCs were gated as CD11c+/ MHC-II high populations, and CD40 expression was measured across conditions (Fig. 10A). Yeasts opsonized with Dectin-1-Fc(IgG2a) induced significantly higher CD40 expression in BMDC (*P* = 0.0001), followed by WGA-Fc(IgG2a) (*P* = 0.0003), compared to control yeasts (Fig. 10B). BMDC activation was further assessed by quantifying IL-6, IL-10, and TNF-α levels in the supernatants (Fig. 10C through E). Treatments with either Lectin-Fc(IgG2a) led to significantly increased secretion of IL-6 (*P* = 0.002 and *P* = 0.0007, respectively; Fig. 10C). IL-10 production was significantly elevated only in cells opsonized with Dectin-1-Fc(IgG2a) (*P* = 0.0007; Fig. 10D). Regarding TNF-α, a trend toward increased production was observed (Fig. 10E).

### Opsonization with Lectin-Fc(IgG2a) fusion proteins resulted in enhanced killing of *C. albicans* by BMDCs

The impact of treatments of *C. albicans* yeasts with Lectin-Fc(IgG2a) proteins on BMDCs phagocytic capacity and fungal viability was also evaluated (Fig. 10F and G). Opsonization with Lectin-Fc(IgGs) did not affect the overall interactions between *C. albicans* and BMDCs (Fig. 10F). Nevertheless, treatments with Lectin-Fc(IgG2a) significantly enhanced BMDC-mediated fungal killing, as evidenced by a reduction in CFUs [Dectin-1-Fc(IgG2a) *P* = 0.0004 and WGA-Fc(IgG2a) *P* = 0.0006; Fig. 10G].

## DISCUSSION

The fungal pathogen *C. albicans* exhibits resistance to most clinically employed antifungal drugs (11). In pursuit of innovative therapeutic alternatives, our group has explored passive immunization strategies using monoclonal antibodies (mAbs) and Lectin-Fc(IgGs) fusion proteins (29–32, 43–47). Herein, we investigated the well-established antifungal direct activity of Lectin-Fc(IgG2a) [including Dectin-1-Fc(IgG2a) and WGA-Fc(IgG2a)] and how they mechanistically affect *C. albicans* structural, physiological, and metabolic functions. We also evaluated their impact on the immune activation of BMDCs, consistent with their previously reported antifungal and immunomodulatory properties (29–31). In the present and previous *in vivo* studies using these Lectin-Fc(IgG) proteins, we included an isotype non-reactive irrelevant mAb as a negative control. Although we understand that we need a pair of non-reactive Lectin-Fc(IgG) to be used as the most appropriate negative control, we are currently in search of a Lectin candidate that demonstrates non-reactivity to any fungal cell wall.

Notably, *C. albicans* can secrete multiple aspartyl proteases (SAPs), recognized virulence factors that play key roles in nutrition, host tissue penetration, and evasion of immune responses, including antibodies and complement (48). Consistent with the importance of the protease activity for fungal escape, despite the growth inhibition in protease-secreting wild-type *C. albicans*, *sap* mutants showed increased susceptibility to Lectin-Fc(IgG2a) treatment. Although SAPs, used as a 20-times concentrated *C. albicans* conditioned supernatant, progressively digested Lectin-Fc(IgG2a) over time, functional proteins remained detectable even after 48 h of incubation in this harsh and ultra-physiological conditions, indicating their stability and robustness, critical aspects for future drug optimization and efficacy assessments. However, future stability and half-life *in vivo* studies will complement the role of fungal or mammalian proteases in degrading these Lectin-Fc during infection.

The fungal cell wall is the main interface between the pathogen and the host immune response. In *C. albicans*, this complex structure consists of an inner layer of chitin, forming the main structural scaffold, β-1,3 and β-1,6-glucans that contribute to rigidity, and an outer fibrillar layer of mannans. The mannans comprising O-glycosylated oligosaccharides and N-glycosylated polysaccharides can mask β-1,3-glucan recognition by Dectin-1, thus interfering with phagocyte-mediated fungal uptake and clearance (49). Disruption of chitin synthesis via deletion of chitin synthases impairs septum formation, cell morphology, and integrity (50) and is associated with compromised permeability and susceptibility to antifungals and host defenses (51). Similarly, disruption of glucan synthase genes (Fks1, Fks2, and Fks3) reduces β-glucan content, altering cell wall composition and reducing growth and viability (12, 49).

In this context, treatments and binding of *C. albicans* with either WGA-Fc(IgG2a) or Dectin-1-Fc(IgG2a) proteins led to significant structural and functional alterations reflecting their antifungal activity, not observed with control non-reacting antibodies. Proteomic enrichment analysis revealed downregulation of proteins involved in cell wall assembly and energy metabolism, including those related to cell surface (GO:0009986), ATP binding (GO:0005524), ADP binding (GO:0043531), and ATPase activity (GO:0016887). Ultrastructural and flow cytometry data confirmed that exposure to Lectin-Fc(IgG2a) compromised cell wall integrity and morphology. TEM revealed a more electron-dense and disorganized cytoplasm, accumulation of intracellular vesicles, and large electron-lucent structures, consistent with stress-induced vesiculation and cellular damage. The surface accessibility of β-1,3-glucan, chitin, and mannosylated residues was significantly reduced, suggesting compromised cell wall integrity and impaired signaling, which contributed to reduced fungal growth and viability. In agreement with our findings, a plant mannose-binding lectin, Helja, either alone or in combination with fluconazole, demonstrated a growth inhibition effect, with direct impact on nuclear integrity, pseudohyphae formation, and cell wall constitution (52).

A major consequence of the Lectin-Fc(IgG2a) binding to *C. albicans* was the induction of oxidative stress, as evidenced by markedly elevated cytoplasmic ROS measured with CM-H2DCFDA (53, 54). This oxidative burst was accompanied by strong upregulation of peroxiredoxin (Q9Y7F0, TSA1), critical for antioxidant responses and redox homeostasis. Similar to our observations, Nabeta et al. (55), demonstrated that an anti-viral mannose-binding lectin, Q-Griffithsin, could inhibit the growth of *Candida* sp. *in vitro*, dramatically impacting the cell wall organization and causing oxidative stress by increasing the intracellular ROS levels. Additionally, enhanced oxidative stress responses have been reported in *C. albicans* treated with antifungals extracted from plant-based essential oils, such as eugenol and citral (from clove and lemon grass, respectively), as well as antimicrobial peptides, such as the protonectin and heat-stable antifungal factor (HSAF), both of which compromise cell membrane integrity and promote apoptosis through ROS accumulation (56–58). In contrast, decreased levels of mitochondrial oxidative enzymes such as succinate-semialdehyde dehydrogenase (Q5AEC3, CAALFM_C303470WA) and reduced superoxide anions (O₂⁻) detected by CellROX-green indicate mitochondrial dysfunction and a compensatory shift in redox balance (59–62). These findings imply that Lectin-Fc(IgG2a) impairs fungal viability by simultaneously compromising cell wall architecture, inducing oxidative and metabolic stress, and suppressing mitochondrial functions. Consistent with these observations, *C. albicans* responds to oxidative stress by inducing chaperones involved in protein folding, proteasome activity, and ribosomal proteins to maintain protein homeostasis (63, 64).

*C. albicans* thrives in various pHs and relies on its metabolic adaptability to colonize host niches (65). In nutrient-limited host environments, *C. albicans* activates pathways that enable glucose production to support survival. Micronutrient homeostasis, particularly involving iron (Fe^3+^), zinc (Zn^2+^), and calcium (Ca^2+^), is essential for enzymatic activity, metabolic processes, and maintaining virulence (11). Calcium, for instance, regulates hyphal thigmotropism and biofilm formation, both of which are critical for tissue invasion, dissemination, and persistence within the host (6, 66). Our data demonstrate that Lectin-Fc(IgG2a) treatment, in addition to inducing metabolic and cell wall structural modifications, disrupts lipid reserves and depletes iron (Fe^3+^), zinc (Zn^2+^), and calcium (Ca^2+^), aligning with the downregulation of ADP/ATP binding protein and impaired energy metabolism. Moreover, micronutrient depletion may further compromise antioxidant defenses and exacerbate the oxidative stress indicated by ROS accumulation, leading to additional cellular stress. Mechanistically, this is consistent with previous findings of Lectin-Fc(IgG2a)-mediated inhibition of *C. albicans* germ tube formation (29, 31) and suppression of *A. fumigatus* germination and biofilm development (30).

Additional effects on cellular homeostasis were evident in the impact of Lectin-Fc(IgG2a) treatment on *C. albicans* EVs release. EVs are spherical bilayered structures carrying proteins, carbohydrates, lipids, and nucleic acids, mediating intercellular communication and interactions with the host (17, 67). Recent studies have demonstrated the EV’s role in yeast-to-hypha differentiation and biofilm formation, confirming that fungal EVs are messengers impacting morphogenesis and virulence (17). Our results showed that Lectin-Fc(IgG2a) treatments negatively affected EV production and their composition, increasing the protein/ergosterol ratio and reducing vesicle size, which could have direct implications in fungal pathogenicity. Similar observations have been reported in *H. capsulatum*, where mAbs targeting Hsp60 increased the protein/sterol ratio in released EVs, which were also smaller and displayed a distinct cargo (68, 69).

DCs are the intersection of innate and adaptive immunity and are key antigen-presenting cells expressing a myriad of pattern recognition receptors (PRRs) for a wide array of pathogen-associated molecular patterns (PAMPs) such as β-glucans, chitins, or other polysaccharides in the fungal cell wall (70). Upon antigen processing, DCs and other antigen-presenting cells (APC) present polypeptides through MHC-II to naive and priming T-cells, which also requires the engagement of costimulatory molecules, such as CD40/CD40L to initiate adaptive responses (71, 72). The enhanced antifungal capacity of BMDCs upon opsonization might help fungal clearance and enhancement of the immune response by promoting the production of cytokines and chemokines that recruit and activate killer cells in infected tissues (25, 73–75). Beyond its direct antifungal effects, Lectin-Fc(IgG2a) enhanced BMDCs activation, increasing CD40 expression and promoting the release of IL-6, IL-10, and TNF-α. The TNF-α production by DCs is correlated with activation and maturation signals, allowing them to better activate naïve T cells (76). On the other hand, activated DCs produce cytokines like IL-12 to drive Th1/Th17 immune responses (76), while IL-6 also promotes migration and T-cell activation (77). *In vivo* studies have corroborated the efficacy of Lectin-Fc(IgG2a) proteins by significantly reducing fungal burden in murine models of histoplasmosis, candidiasis (including *C. auris* and *C. albicans*), cryptococcosis, and aspergillosis (29–32). These reductions in fungal burden coincide with a decrease in lung injury, as evidenced by elevated IFN-γ, reduced IL-4 and IL-10 levels (29), ultimately resulting in improved survival of the infected animals following treatment.

In summary, our findings demonstrate that Lectin-Fc(IgG2a) proteins target *C. albicans* through multiple complementary mechanisms, including disrupting the cell wall structure, metabolic shift, micronutrient depletion, oxidative stress induction, interference with EV biogenesis, and host immunomodulation. Therefore, these diverse effects of Lectin-Fc(IgG2a) act synergistically to eliminate *C. albicans* and enhance host defenses, as previously demonstrated in several fungal models, further supporting their potential as pan-antifungal immunobiologicals and their development as promising alternatives to conventional antifungals. We understand that additional stability studies need to be performed *in vivo*, including potentially protease-rich environments or in combination with protease inhibitors, which could enhance their efficacy. Additionally, evaluations of potential off-target binding and immunogenicity need to be addressed before considering the application of engineering strategies for their humanization and future translational studies to human subjects.

## Data Availability

The mass spectrometry proteomics data have been deposited to the ProteomeXchange Consortium PRIDE (accession number PXD067198) (39).

## SUPPLEMENTAL MATERIAL

Fig. S1 spectrum.03645-25-s0001.tif *C. albicans* proteases mediate digestion of the Lectin-Fc(IgG2a) proteins.

Fig. S2 spectrum.03645-25-s0002.tif STRING protein interaction network of upregulated and downregulated proteins upon treatment of *C. albicans* yeasts with Lectin-Fc(IgG2a).

Fig. S3 spectrum.03645-25-s0003.tif KEGG pathway enrichment analysis demonstrating the proteins participating in glycolysis/gluconeogenesis and metabolism of amino acids, with lower abundance (green plots) and higher abundance (red plots) upon co-incubations with Lectin-Fc(IgG2a).

Fig. S4 spectrum.03645-25-s0004.tif Immunofluorescence of *C. albicans* yeasts displaying the contents of cell wall polysaccharides upon treatment with the Lectin-Fc(IgG2a).

Table S1 spectrum.03645-25-s0005.xlsx Proteomic analysis of *C. albicans* yeasts upon treatment with Lectin-Fc(IgG2a) [Dectin-1-Fc(IgG2a) and WGA-Fc(IgG2a)].

ASM does not own the copyrights to Supplemental Material that may be linked to, or accessed through, an article. The authors have granted ASM a non-exclusive, world-wide license to publish the Supplemental Material files. Please contact the corresponding author directly for reuse.

## References

[1] Denning DW. 2024. Renaming Candida glabrata-A case of taxonomic purity over clinical and public health pragmatism. PLoS Pathog 20:e1012055. doi:10.1371/journal.ppat.1012055 38489254 PMC10942050

[2] Borman AM, Abdolrasouli A, Johnson EM. 2025. Name changes for fungi of medical importance, 2022-2024. J Clin Microbiol 63:e0204124. doi:10.1128/jcm.02041-24 40899797 PMC12607852

[3] McCarty TP, White CM, Pappas PG. 2021. Candidemia and invasive candidiasis. Infect Dis Clin North Am 35:389–413. doi:10.1016/j.idc.2021.03.007 34016283

[4] Liu F, Hu Z-D, Zhao X-M, Zhao W-N, Feng Z-X, Yurkov A, Alwasel S, Boekhout T, Bensch K, Hui F-L, Bai F-Y, Wang Q-M. 2024. Phylogenomic analysis of the Candida auris-Candida haemuli clade and related taxa in the Metschnikowiaceae, and proposal of thirteen new genera, fifty-five new combinations and nine new species. Persoonia 52:22–43. doi:10.3767/persoonia.2024.52.02 39161632 PMC11319837

[5] Spivak ES, Hanson KE. 2018. Candida auris: an emerging fungal pathogen. J Clin Microbiol 56:e01588-17. doi:10.1128/JCM.01588-17 29167291 PMC5786713

[6] Talapko J, Juzbašić M, Matijević T, Pustijanac E, Bekić S, Kotris I, Škrlec I. 2021. Candida albicans-the virulence factors and clinical manifestations of infection. J Fungi (Basel) 7:79. doi:10.3390/jof7020079 33499276 PMC7912069

[7] Kilpatrick R, Scarrow E, Hornik C, Greenberg RG. 2022. Neonatal invasive candidiasis: updates on clinical management and prevention. Lancet Child Adolesc Health 6:60–70. doi:10.1016/S2352-4642(21)00272-8 34672994

[8] Andes DR, Safdar N, Baddley JW, Alexander B, Brumble L, Freifeld A, Hadley S, Herwaldt L, Kauffman C, Lyon GM, Morrison V, Patterson T, Perl T, Walker R, Hess T, Chiller T, Pappas PG, The TRANSNET Investigators. 2016. The epidemiology and outcomes of invasive Candida infections among organ transplant recipients in the United States: results of the Transplant‐Associated Infection Surveillance Network (TRANSNET). Transplant Infectious Dis 18:921–931. doi:10.1111/tid.12613 27643395

[9] Dadar M, Tiwari R, Karthik K, Chakraborty S, Shahali Y, Dhama KCA. 2018. Candida albicans - biology, molecular characterization, pathogenicity, and advances in diagnosis and control - an update. Microb Pathog 117:128–138. doi:10.1016/j.micpath.2018.02.028 29454824

[10] Steinbach WJ. 2016. Pediatric invasive candidiasis: epidemiology and diagnosis in children. J Fungi (Basel) 2:5. doi:10.3390/jof2010005 29376923 PMC5753086

[11] Wijnants S, Vreys J, Van Dijck P. 2022. Interesting antifungal drug targets in the central metabolism of Candida albicans. Trends Pharmacol Sci 43:69–79. doi:10.1016/j.tips.2021.10.003 34756759

[12] Arita GS, Faria DR, Capoci IRG, Kioshima ES, Bonfim-Mendonça PS, Svidzinski TIE. 2022. Cell wall associated proteins involved in filamentation with impact on the virulence of Candida albicans. Microbiol Res 258:126996. doi:10.1016/j.micres.2022.126996 35247799

[13] Kean R, Ramage G. 2019. Combined antifungal resistance and biofilm tolerance: the global threat of Candida auris. mSphere 4:e00458-19. doi:10.1128/mSphere.00458-19 31366705 PMC6669339

[14] Pereira R, Dos Santos Fontenelle RO, de Brito EHS, de Morais SM. 2021. Biofilm of Candida albicans: formation, regulation and resistance. J Appl Microbiol 131:11–22. doi:10.1111/jam.14949 33249681

[15] de Toledo Martins S, Szwarc P, Goldenberg S, Alves LR. 2019. Extracellular vesicles in fungi: composition and functions. Curr Top Microbiol Immunol 422:45–59. doi:10.1007/82_2018_141 30242512

[16] Rizzo J, Rodrigues ML, Janbon G. 2020. Extracellular vesicles in fungi: past, present, and future perspectives. Front Cell Infect Microbiol 10:346. doi:10.3389/fcimb.2020.00346 32760680 PMC7373726

[17] Honorato L, de Araujo JFD, Ellis CC, Piffer AC, Pereira Y, Frases S, de Sousa Araújo GR, Pontes B, Mendes MT, Pereira MD, Guimarães AJ, da Silva NM, Vargas G, Joffe L, Del Poeta M, Nosanchuk JD, Zamith-Miranda D, Dos Reis FCG, de Oliveira HC, Rodrigues ML, de Toledo Martins S, Alves LR, Almeida IC, Nimrichter L. 2022. Extracellular vesicles regulate biofilm formation and yeast-to-hypha differentiation in Candida albicans. mBio 13:e0030122. doi:10.1128/mbio.00301-22 35420476 PMC9239257

[18] Rodrigues ML, Franzen AJ, Nimrichter L, Miranda K. 2013. Vesicular mechanisms of traffic of fungal molecules to the extracellular space. Curr Opin Microbiol 16:414–420. doi:10.1016/j.mib.2013.04.002 23628115

[19] Vargas G, Rocha JDB, Oliveira DL, Albuquerque PC, Frases S, Santos SS, Nosanchuk JD, Gomes AMO, Medeiros LCAS, Miranda K, Sobreira TJP, Nakayasu ES, Arigi EA, Casadevall A, Guimaraes AJ, Rodrigues ML, Freire-de-Lima CG, Almeida IC, Nimrichter L. 2015. Compositional and immunobiological analyses of extracellular vesicles released by Candida albicans. Cell Microbiol 17:389–407. doi:10.1111/cmi.12374 25287304

[20] Van Rhijn N, White PL. 2025. Antifungal treatment strategies and their impact on resistance development in clinical settings. J Antimicrob Chemother 80:3208–3226. doi:10.1093/jac/dkaf382 41092294 PMC12670168

[21] Liu D, Zhou R, Gao X. 2025. Recent innovations and challenges in the treatment of fungal infections. Front Cell Infect Microbiol 15:1676009. doi:10.3389/fcimb.2025.1676009 41104137 PMC12521125

[22] Maubon D, Garnaud C, Calandra T, Sanglard D, Cornet M. 2014. Resistance of Candida spp. to antifungal drugs in the ICU: where are we now? Intensive Care Med 40:1241–1255. doi:10.1007/s00134-014-3404-7 25091787

[23] Arendrup MC, Patterson TF. 2017. Multidrug-resistant Candida: epidemiology, molecular mechanisms, and treatment. J Infect Dis 216:S445–S451. doi:10.1093/infdis/jix131 28911043

[24] Paiva J-A, Pereira JM. 2023. Treatment of invasive candidiasis in the era of Candida resistance. Curr Opin Crit Care 29:457–462. doi:10.1097/MCC.0000000000001077 37641511

[25] Pappas PG, Lionakis MS, Arendrup MC, Ostrosky-Zeichner L, Kullberg BJ. 2018 Invasive candidiasis. Nat Rev Dis Primers 4:18026. doi:10.1038/nrdp.2018.26 29749387

[26] Boniche C, Rossi SA, Kischkel B, Barbalho FV, Moura ÁND, Nosanchuk JD, Travassos LR, Taborda CP. 2020. Immunotherapy against systemic fungal infections based on monoclonal antibodies. J Fungi (Basel) 6:31. doi:10.3390/jof6010031 32121415 PMC7151209

[27] Ulrich S, Ebel F. 2020. Monoclonal antibodies as tools to combat fungal infections. J Fungi (Basel) 6:22. doi:10.3390/jof6010022 32033168 PMC7151206

[28] Gow NAR, Latge JP, Munro CA. 2017. The fungal cell wall: structure, biosynthesis, and function. Microbiol Spectr 5:5. doi:10.1128/microbiolspec.funk-0035-2016 PMC11687499 28513415

[29] Liedke SC, Miranda DZ, Gomes KX, Gonçalves JLS, Frases S, Nosanchuk JD, Rodrigues ML, Nimrichter L, Peralta JM, Guimarães AJ. 2017. Characterization of the antifungal functions of a WGA-Fc (IgG2a) fusion protein binding to cell wall chitin oligomers. Sci Rep 7:12187. doi:10.1038/s41598-017-12540-y 28939893 PMC5610272

[30] Rodriguez-de la Noval C, Ruiz Mendoza S, de Souza Gonçalves D, da Silva Ferreira M, Honorato L, Peralta JM, Nimrichter L, Guimarães AJ. 2020. Protective efficacy of lectin-Fc(IgG) fusion proteins in vitro and in a pulmonary aspergillosis in vivo mode. J Fungi 6:250. doi:10.3390/jof6040250 PMC7712007 33120893

[31] Ruiz Mendoza S, Liedke SC, Rodriguez de La Noval C, Ferreira M da S, Gomes KX, Honorato L, Nimrichter L, Peralta JM, Guimarães AJ. 2022. In vitro and in vivo efficacies of dectin-1-Fc(IgG)(s) fusion proteins against invasive fungal infections. Med Mycol 60:myac050. doi:10.1093/mmy/myac050 35867978

[32] Mendoza SR, Honorato L, Cintra DS, Ferreira M da S, Zamith-Miranda D, Nosanchuk JD, Nimrichter L, Guimarães AJ. 2025. Lectin-Fc(IgG) fusion proteins exhibit antifungal activity against the emerging multidrug-resistant pathogen Candida auris. Infect Immun 93:e0032925. doi:10.1128/iai.00329-25 41159718 PMC12707105

[33] Hube B, Sanglard D, Odds FC, Hess D, Monod M, Schäfer W, Brown AJ, Gow NA. 1997. Disruption of each of the secreted aspartyl proteinase genes SAP1, SAP2, and SAP3 of Candida albicans attenuates virulence. Infect Immun 65:3529–3538. doi:10.1128/iai.65.9.3529-3538.1997 9284116 PMC175503

[34] Ibrahim AS, Filler SG, Sanglard D, Edwards JE, Hube B. 1998. Secreted aspartyl proteinases and interactions of Candida albicans with human endothelial cells. Infect Immun 66:3003–3005. doi:10.1128/IAI.66.6.3003-3005.1998 9596782 PMC108304

[35] Sanglard D, Hube B, Monod M, Odds FC, Gow NA. 1997. A triple deletion of the secreted aspartyl proteinase genes SAP4, SAP5, and SAP6 of Candida albicans causes attenuated virulence. Infect Immun 65:3539–3546. doi:10.1128/iai.65.9.3539-3546.1997 9284117 PMC175504

[36] Cordero RJB, Liedke SC, de S Araújo GR, Martinez LR, Nimrichter L, Frases S, Peralta JM, Casadevall A, Rodrigues ML, Nosanchuk JD, Guimaraes AJ. 2016. Enhanced virulence of Histoplasma capsulatum through transfer and surface incorporation of glycans from Cryptococcus neoformans during co-infection. Sci Rep 6:21765. doi:10.1038/srep21765 26908077 PMC4764860

[37] Hu X, Yang P, Chai C, Liu J, Sun H, Wu Y, Zhang M, Zhang M, Liu X, Yu H. 2023. Structural and mechanistic insights into fungal β-1,3-glucan synthase FKS1. Nature 616:190–198. doi:10.1038/s41586-023-05856-5 36949198 PMC10032269

[38] Cox J, Mann M. 2008. MaxQuant enables high peptide identification rates, individualized p.p.b.-range mass accuracies and proteome-wide protein quantification. Nat Biotechnol 26:1367–1372. doi:10.1038/nbt.1511 19029910

[39] Perez-Riverol Y, Csordas A, Bai J, Bernal-Llinares M, Hewapathirana S, Kundu DJ, Inuganti A, Griss J, Mayer G, Eisenacher M, Pérez E, Uszkoreit J, Pfeuffer J, Sachsenberg T, Yilmaz S, Tiwary S, Cox J, Audain E, Walzer M, Jarnuczak AF, Ternent T, Brazma A, Vizcaíno JA. 2019. The PRIDE database and related tools and resources in 2019: improving support for quantification data. Nucleic Acids Res 47:D442–D450. doi:10.1093/nar/gky1106 30395289 PMC6323896

[40] Roney K. 2019. Bone marrow-derived dendritic cells. Methods Mol Biol 1960:57–62. doi:10.1007/978-1-4939-9167-9_4 30798520

[41] Elderman M, van Beek A, Brandsma E, de Haan B, Savelkoul H, de Vos P, Faas M. 2016. Sex impacts Th1 cells, Tregs, and DCs in both intestinal and systemic immunity in a mouse strain and location-dependent manner. Biol Sex Differ 7:21. doi:10.1186/s13293-016-0075-9 27051505 PMC4820953

[42] Ferreira M da S, Mendoza SR, Gonçalves D de S, Rodríguez-de la Noval C, Honorato L, Nimrichter L, Ramos LFC, Nogueira FCS, Domont GB, Peralta JM, Guimarães AJ. 2022. Recognition of cell wall mannosylated components as a conserved feature for fungal entrance, adaptation and survival within trophozoites of Acanthamoeba castellanii and murine macrophages. Front Cell Infect Microbiol 12:858979. doi:10.3389/fcimb.2022.858979 35711659 PMC9194641

[43] Guimarães AJ, Hamilton AJ, de M Guedes HL, Nosanchuk JD, Zancopé-Oliveira RM. 2008. Biological function and molecular mapping of M antigen in yeast phase of Histoplasma capsulatum. PLoS One 3:e3449. doi:10.1371/journal.pone.0003449 18927619 PMC2566600

[44] Guimarães AJ, Frases S, Gomez FJ, Zancopé-Oliveira RM, Nosanchuk JD. 2009. Monoclonal antibodies to heat shock protein 60 alter the pathogenesis of Histoplasma capsulatum. Infect Immun 77:1357–1367. doi:10.1128/IAI.01443-08 19179416 PMC2663142

[45] Guimarães AJ, Frases S, Pontes B, de Cerqueira MD, Rodrigues ML, Viana NB, Nimrichter L, Nosanchuk JD. 2011. Agglutination of Histoplasma capsulatum by IgG monoclonal antibodies against Hsp60 impacts macrophage effector functions. Infect Immun 79:918–927. doi:10.1128/IAI.00673-10 21134968 PMC3028823

[46] Guimarães AJ, Martinez LR, Nosanchuk JD. 2011. Passive administration of monoclonal antibodies against H. capsulatum and other fungal pathogens. J Vis Exp 2532:2532. doi:10.3791/2532 PMC3197409 21372781

[47] Lopes LCL, Guimarães AJ, de Cerqueira MD, Gómez BL, Nosanchuk JD. 2010. A Histoplasma capsulatum-specific IgG1 isotype monoclonal antibody, H1C, to a 70-kilodalton cell surface protein is not protective in murine histoplasmosis. Clin Vaccine Immunol 17:1155–1158. doi:10.1128/CVI.00033-10 20484567 PMC2897255

[48] Naglik JR, Challacombe SJ, Hube B. 2003. Candida albicans secreted aspartyl proteinases in virulence and pathogenesis. Microbiol Mol Biol Rev 67:400–428. doi:10.1128/MMBR.67.3.400-428.2003 12966142 PMC193873

[49] Garcia-Rubio R, de Oliveira HC, Rivera J, Trevijano-Contador N. 2019. The fungal cell wall: Candida, Cryptococcus, and Aspergillus species. Front Microbiol 10:2993. doi:10.3389/fmicb.2019.02993 31993032 PMC6962315

[50] Munro CA, Winter K, Buchan A, Henry K, Becker JM, Brown AJP, Bulawa CE, Gow NAR. 2004. Chs1 of Candida albicans is an essential chitin synthase required for synthesis of the septum and for cell integrity: CaChs1p is essential for growth and septum formation. Mol Microbiol 39:1414–1426. doi:10.1046/j.1365-2958.2001.02347.x 11251855

[51] Zhang SQ, Zou Z, Shen H, Shen SS, Miao Q, Huang X, Liu W, Li LP, Chen SM, Yan L, Zhang JD, Zhao JJ, Xu GT, An MM, Jiang YY. 2016. Mnn10 maintains pathogenicity in Candida albicans by extending α-1,6-mannose backbone to evade host dectin-1 mediated antifungal immunity. PLoS Pathog 12:e1005617. doi:10.1371/journal.ppat.1005617 27144456 PMC4856274

[52] Del Rio M, Radicioni MB, Mello ÉO, Ribeiro SFF, Taveira GB, Carvalho AO, de la Canal L, Gomes VM, Regente M. 2022. A plant mannose-binding lectin and fluconazole: key targets combination against Candida albicans. J Appl Microbiol 132:4310–4320. doi:10.1111/jam.15544 35332971

[53] Akter S, Khan MS, Smith EN, Flashman E. 2021. Measuring ROS and redox markers in plant cells. RSC Chem Biol 2:1384–1401. doi:10.1039/d1cb00071c 34704044 PMC8495998

[54] Oparka M, Walczak J, Malinska D, van Oppen LMPE, Szczepanowska J, Koopman WJH, Wieckowski MR. 2016. Quantifying ROS levels using CM-H 2 DCFDA and HyPer. Methods 109:3–11. doi:10.1016/j.ymeth.2016.06.008 27302663

[55] Nabeta HW, Kouokam JC, Lasnik AB, Fuqua JL, Palmer KE. 2021. Novel antifungal activity of Q-griffithsin, a broad-spectrum antiviral lectin. Microbiol Spectr 9:e0095721. doi:10.1128/Spectrum.00957-21 34494857 PMC8557872

[56] Ding Y, Li Z, Li Y, Lu C, Wang H, Shen Y, Du L. 2016. HSAF-induced antifungal effects in Candida albicans through ROS-mediated apoptosis. RSC Adv 6:30895–30904. doi:10.1039/C5RA26092B 27594989 PMC5006743

[57] Shahina Z, Ndlovu E, Persaud O, Sultana T, Dahms TES. 2022. Candida albicans reactive oxygen species (ROS)-dependent lethality and ros-independent hyphal and biofilm inhibition by eugenol and citral. Microbiol Spectr 10:e0318322. doi:10.1128/spectrum.03183-22 36394350 PMC9769929

[58] Wang K, Dang W, Xie J, Zhu R, Sun M, Jia F, Zhao Y, An X, Qiu S, Li X, Ma Z, Yan W, Wang R. 2015. Antimicrobial peptide protonectin disturbs the membrane integrity and induces ROS production in yeast cells. Biochim Biophys Acta 1848:2365–2373. doi:10.1016/j.bbamem.2015.07.008 26209560

[59] Deragon MA, McCaig WD, Patel PS, Haluska RJ, Hodges AL, Sosunov SA, Murphy MP, Ten VS, LaRocca TJ. 2020. Mitochondrial ROS prime the hyperglycemic shift from apoptosis to necroptosis. Cell Death Discov 6:132. doi:10.1038/s41420-020-00370-3 33298902 PMC7693268

[60] Jitschin R, Hofmann AD, Bruns H, Giessl A, Bricks J, Berger J, Saul D, Eckart MJ, Mackensen A, Mougiakakos D. 2014. Mitochondrial metabolism contributes to oxidative stress and reveals therapeutic targets in chronic lymphocytic leukemia. Blood 123:2663–2672. doi:10.1182/blood-2013-10-532200 24553174

[61] Yokoyama C, Sueyoshi Y, Ema M, Mori Y, Takaishi K, Hisatomi H. 2017. Induction of oxidative stress by anticancer drugs in the presence and absence of cells. Oncol Lett 14:6066–6070. doi:10.3892/ol.2017.6931 29113247 PMC5661396

[62] Yu Q, Zhang B, Li J, Zhang B, Wang H, Li M. 2016. Endoplasmic reticulum-derived reactive oxygen species (ROS) is involved in toxicity of cell wall stress to Candida albicans. Free Radic Biol Med 99:572–583. doi:10.1016/j.freeradbiomed.2016.09.014 27650297

[63] Amador-García A, Zapico I, Borrajo A, Malmström J, Monteoliva L, Gil C. 2021. Extending the proteomic characterization of Candida albicans exposed to stress and apoptotic inducers through data-independent acquisition mass spectrometry. mSystems 6:e0094621. doi:10.1128/mSystems.00946-21 34609160 PMC8547427

[64] Dantas A da S, Day A, Ikeh M, Kos I, Achan B, Quinn J. 2015. Oxidative stress responses in the human fungal pathogen, Candida albicans. Biomolecules 5:142–165. doi:10.3390/biom5010142 25723552 PMC4384116

[65] Brown AJP, Odds FC, Gow NAR. 2007. Infection-related gene expression in Candida albicans. Curr Opin Microbiol 10:307–313. doi:10.1016/j.mib.2007.04.001 17707687

[66] Li W, Shrivastava M, Lu H, Jiang Y. 2021. Calcium-calcineurin signaling pathway in Candida albicans: a potential drug target. Microbiol Res 249:126786. doi:10.1016/j.micres.2021.126786 33989979

[67] Wei Y, Wang Z, Liu Y, Liao B, Zong Y, Shi Y, Liao M, Wang J, Zhou X, Cheng L, Ren B. 2023. Extracellular vesicles of Candida albicans regulate its own growth through the l-arginine/nitric oxide pathway. Appl Microbiol Biotechnol 107:355–367. doi:10.1007/s00253-022-12300-7 36441207 PMC9703431

[68] Baltazar LM, Zamith-Miranda D, Burnet MC, Choi H, Nimrichter L, Nakayasu ES, Nosanchuk JD. 2018. Concentration-dependent protein loading of extracellular vesicles released by Histoplasma capsulatum after antibody treatment and its modulatory action upon macrophages. Sci Rep 8:8065. doi:10.1038/s41598-018-25665-5 29795301 PMC5966397

[69] Matos Baltazar L, Nakayasu ES, Sobreira TJP, Choi H, Casadevall A, Nimrichter L, Nosanchuk JD. 2016. Antibody binding alters the characteristics and contents of extracellular vesicles released by Histoplasma capsulatum. mSphere 1:e00085-15. doi:10.1128/mSphere.00085-15 PMC4894687 27303729

[70] Ramirez-Ortiz ZG, Means TK. 2012. The role of dendritic cells in the innate recognition of pathogenic fungi (A. fumigatus, C. neoformans and C. albicans). Virulence 3:635–646. doi:10.4161/viru.22295 23076328 PMC3545945

[71] Elgueta R, Benson MJ, de Vries VC, Wasiuk A, Guo Y, Noelle RJ. 2009. Molecular mechanism and function of CD40/CD40L engagement in the immune system. Immunol Rev 229:152–172. doi:10.1111/j.1600-065X.2009.00782.x 19426221 PMC3826168

[72] Iwasaki A, Medzhitov R. 2015. Control of adaptive immunity by the innate immune system. Nat Immunol 16:343–353. doi:10.1038/ni.3123 25789684 PMC4507498

[73] Erwig LP, Gow NAR. 2016. Interactions of fungal pathogens with phagocytes. Nat Rev Microbiol 14:163–176. doi:10.1038/nrmicro.2015.21 26853116

[74] Netea MG, Gijzen K, Coolen N, Verschueren I, Figdor C, Van der Meer JWM, Torensma R, Kullberg BJ. 2004. Human dendritic cells are less potent at killing Candida albicans than both monocytes and macrophages. Microbes Infect 6:985–989. doi:10.1016/j.micinf.2004.05.013 15345229

[75] Roy RM, Klein BS. 2012. Dendritic cells in antifungal immunity and vaccine design. Cell Host Microbe 11:436–446. doi:10.1016/j.chom.2012.04.005 22607797 PMC3401965

[76] Blanco P, Palucka AK, Pascual V, Banchereau J. 2008. Dendritic cells and cytokines in human inflammatory and autoimmune diseases. Cytokine Growth Factor Rev 19:41–52. doi:10.1016/j.cytogfr.2007.10.004 18258476 PMC2413068

[77] Xu Y-D, Cheng M, Shang P-P, Yang Y-Q. 2022. Role of IL-6 in dendritic cell functions. J Leukoc Biol 111:695–709. doi:10.1002/JLB.3MR0621-616RR 34405445

