# Supplementary figures and images for: Lectin-Fc(IgG2a) fusion proteins binding to the cell wall of C. albicans cause structural, metabolic, oxidative, and other pleiotropic effects
Source: Microbiol Spectr. 2026 May 14;14(6):e03645-25. doi: 10.1128/spectrum.03645-25 (PMC13227963; doi:10.1128/spectrum.03645-25)

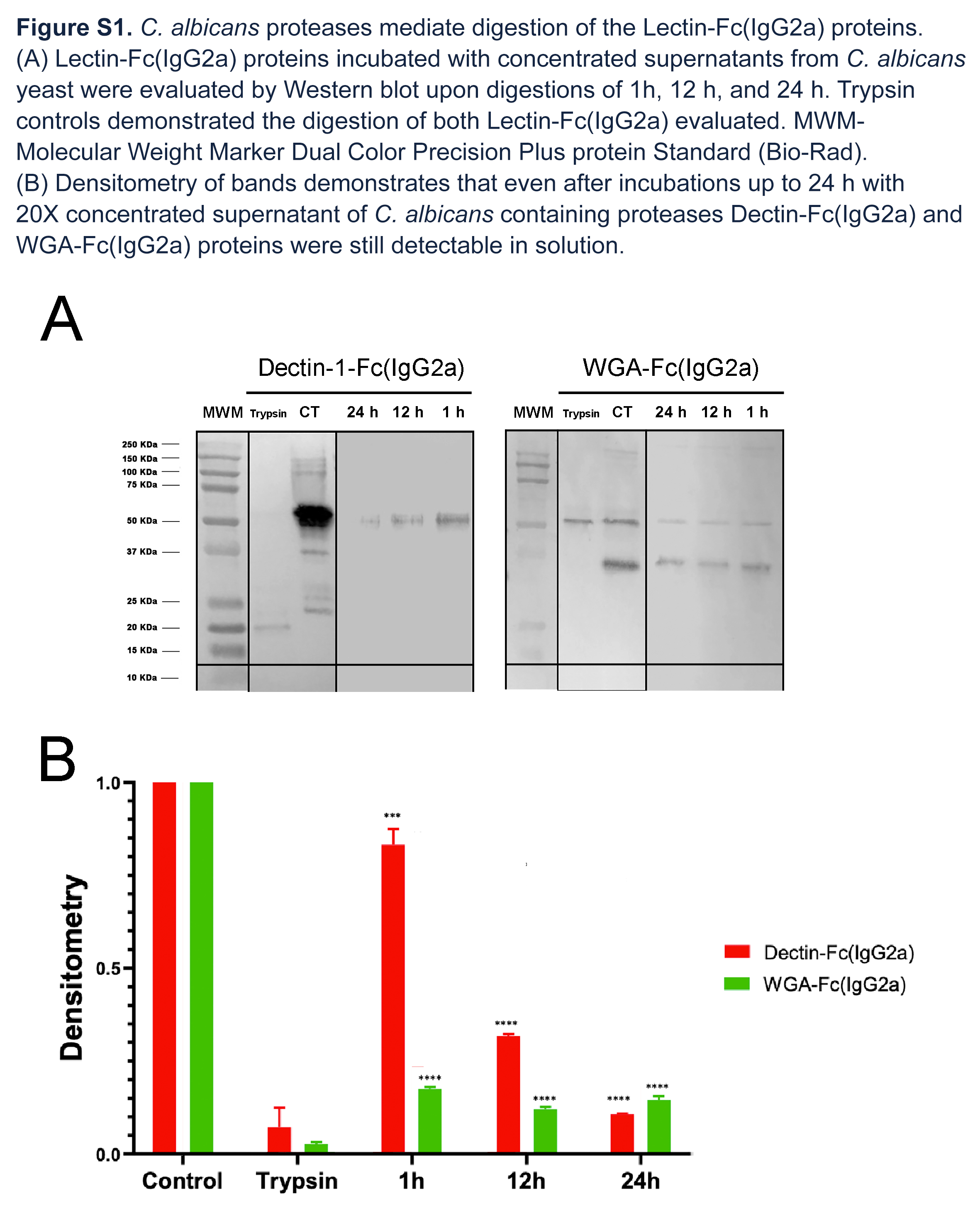

Supplement: Fig. S1 — C. albicans proteases mediate digestion of the Lectin-Fc(IgG2a) proteins. [file spectrum.03645-25-s0001.tif]

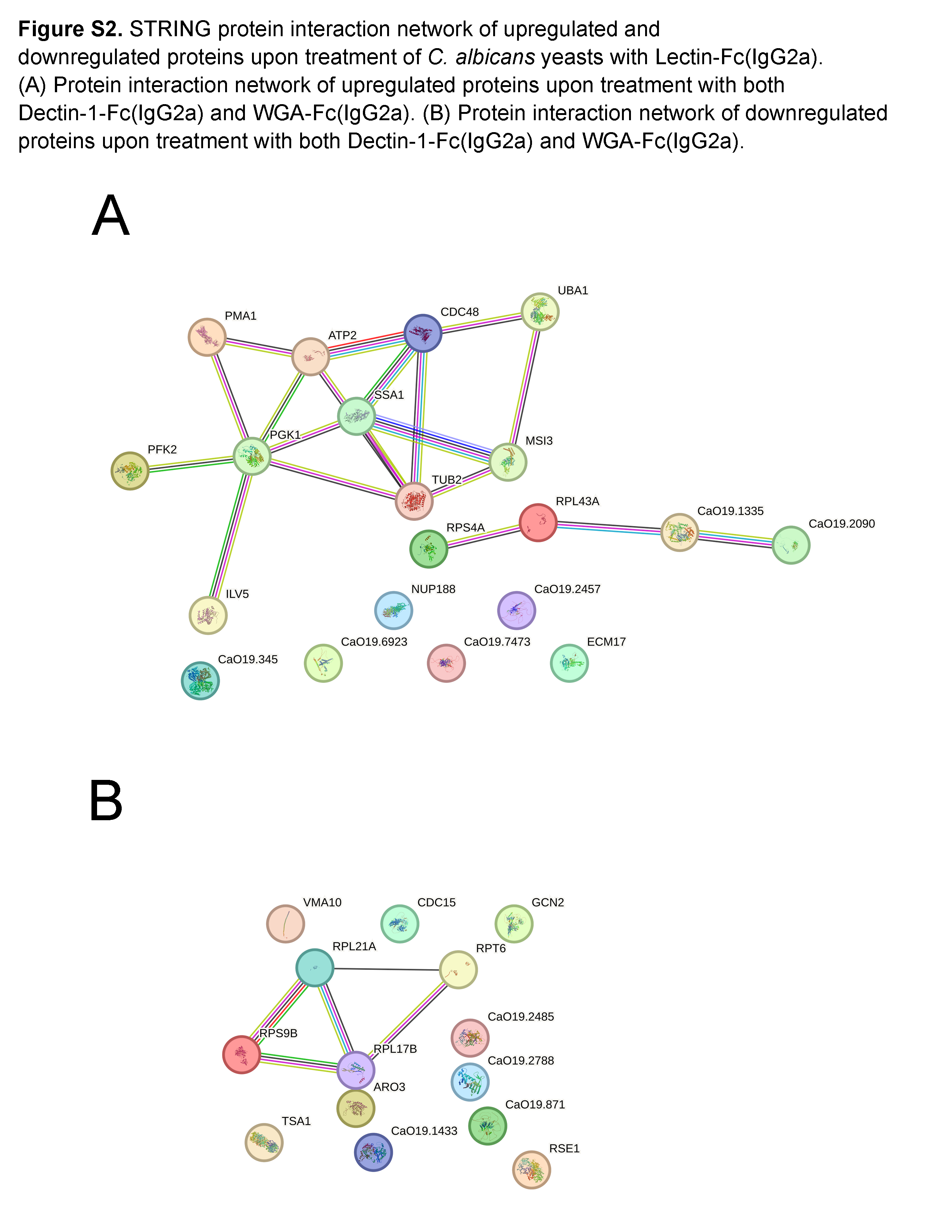

Supplement: Fig. S2 — STRING protein interaction network of upregulated and downregulated proteins upon treatment of C. albicans yeasts with Lectin-Fc(IgG2a). [file spectrum.03645-25-s0002.tif]

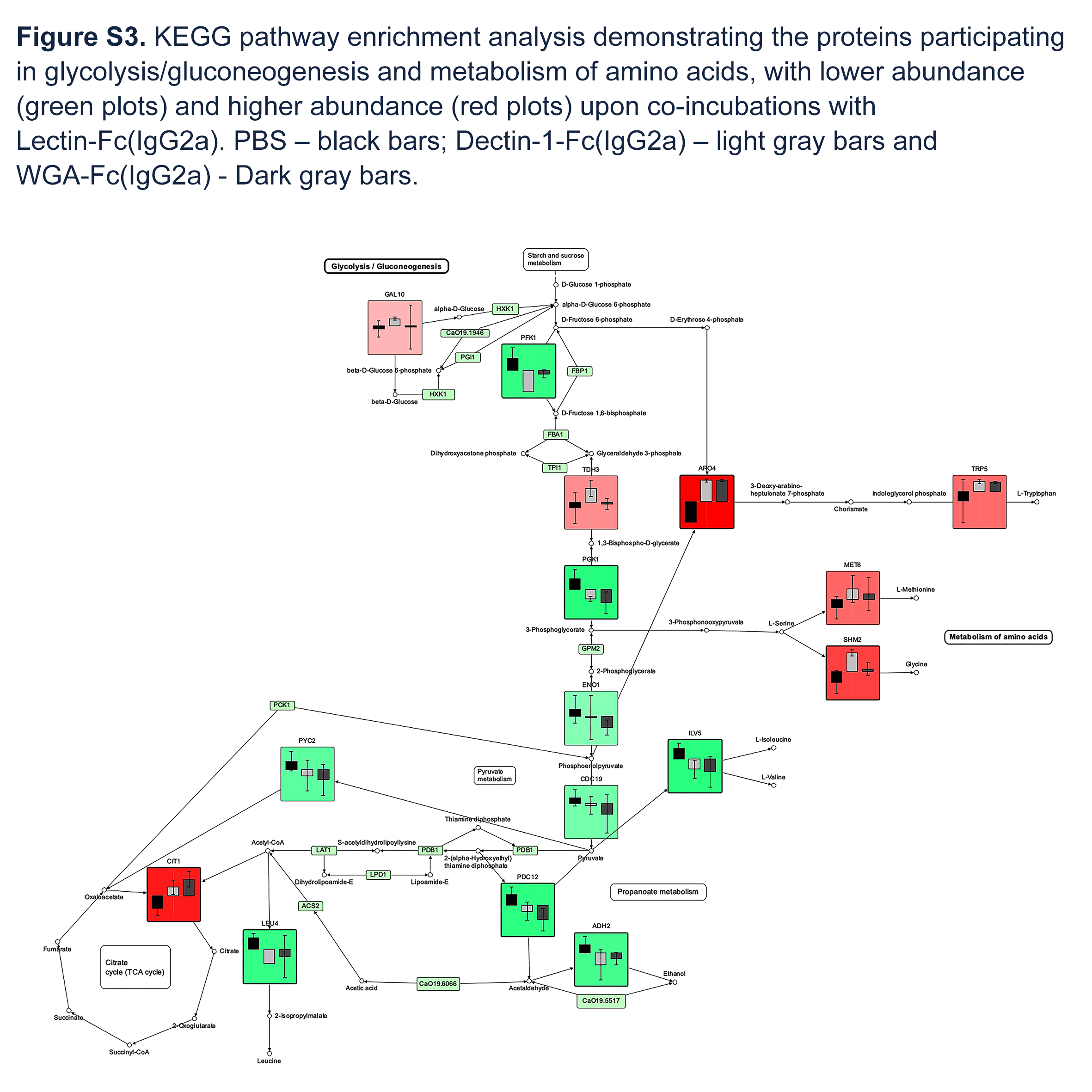

Supplement: Fig. S3 — KEGG pathway enrichment analysis demonstrating the proteins participating in glycolysis/gluconeogenesis and metabolism of amino acids, with lower abundance (green plots) and higher abundance (red plots) upon co-incubations with Lectin-Fc(IgG2a). [file spectrum.03645-25-s0003.tif]

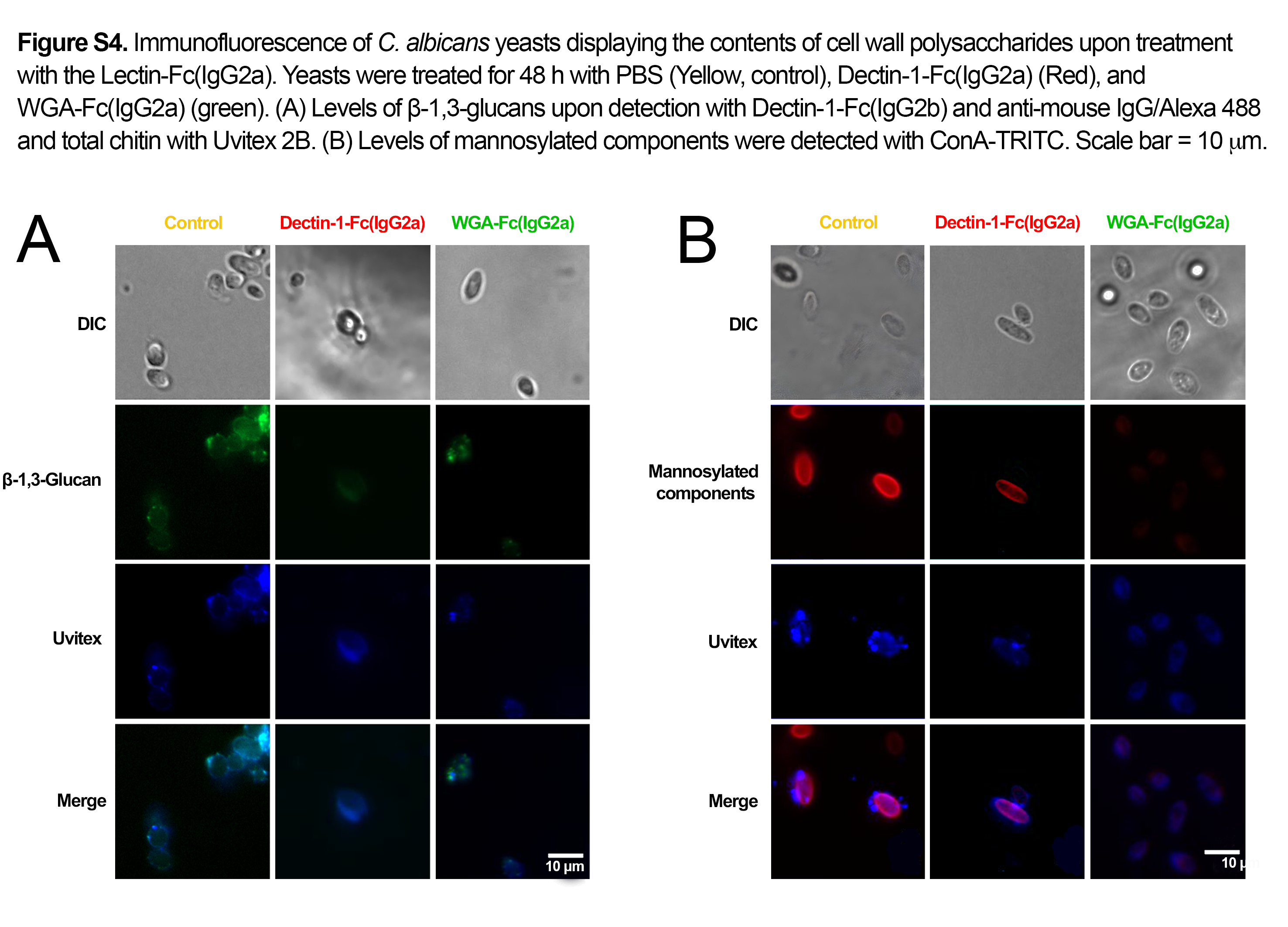

Supplement: Fig. S4 — Immunofluorescence of C. albicans yeasts displaying the contents of cell wall polysaccharides upon treatment with the Lectin-Fc(IgG2a). [file spectrum.03645-25-s0004.tif]
